# Biologically inspired heterogeneous learning for accurate, efficient and low-latency neural network

**DOI:** 10.1093/nsr/nwae301

**Published:** 2024-08-30

**Authors:** Bo Wang, Yuxuan Zhang, Hongjue Li, Hongkun Dou, Yuchen Guo, Yue Deng

**Affiliations:** School of Astronautics, Beihang University, Beijing 100191, China; School of Astronautics, Beihang University, Beijing 100191, China; School of Astronautics, Beihang University, Beijing 100191, China; School of Astronautics, Beihang University, Beijing 100191, China; Institute for Brain and Cognitive Sciences, BNRist, Tsinghua University, Beijing 100084, China; School of Astronautics, Beihang University, Beijing 100191, China; School of Artificial Intelligence, Beihang University, Beijing 100191, China

**Keywords:** spiking neural network, memory, heterogeneous learning, biologically inspired, scRNA-seq

## Abstract

The pursuit of artificial neural networks that mirror the accuracy, efficiency and low latency of biological neural networks remains a cornerstone of artificial intelligence (AI) research. Here, we incorporated recent neuroscientific findings of self-inhibiting autapse and neuron heterogeneity for innovating a spiking neural network (SNN) with enhanced learning and memorizing capacities. A bi-level programming paradigm was formulated to respectively learn neuron-level biophysical variables and network-level synapse weights for nested heterogeneous learning. We successfully demonstrated that our biologically inspired neuron model could reproduce neural statistics at both individual and group levels, contributing to the effective decoding of brain–computer interface data. Furthermore, the heterogeneous SNN showed higher accuracy (1%–10% improvement), superior efficiency (maximal 17.83-fold reduction in energy) and lower latency (maximal 5-fold improvement) in performing several AI tasks. For the first time, we benchmarked SNN for conducting cell type identification from scRNA-seq data. The proposed model correctly identified very rare cell types associated with severe brain diseases where typical SNNs failed.

## Teaser

Incorporating self-inhibiting autapse into spiking neurons, we propose a neuron-level memory network, and hierarchical learning framework to optimize neuron-level and network-level variables, yielding a heterogeneous SNN with enhanced learning capabilities.

## INTRODUCTION

Compactly structured neural networks, formed by billions of heterogeneous nerve cells, make our brain a marvelous system in nature [1]. These nerve cells communicate with each other through synaptic transmission in the form of spikes, granting the effective and efficient cognition ability to human brains [2]. In the interdisciplinary areas of artificial intelligence (AI) and neuroscience, there has been long-term pursuit for brain-inspired AI models that can approach the performance of the biological brain. As the most influential brain-inspired models, analog-valued neural networks (ANNs) achieve sound performance in accuracy but sacrifice energy efficiency and inference latency [3]. Alternatively, spiking neural networks (SNNs) follow a more biologically inspired way to directly characterize the spiking mechanisms of nerve cells, offering great potential to accomplish accurate, efficient and low-latency machine intelligence [4].

In AI, various SNN models have been configured with alternative inspirations from biology, physics or computer science. The integrate-and-fire (IF) neuron [5] explicitly models the spike formation process in terms of membrane potential thresholding. The leaky IF (LIF) neuron [6] introduces the decay factor into IF to better approximate the degradation of membrane potential at different time steps. The spike response model (SRM) [7] enhances the nonlinearity of the spiking neuron via kernel tricks. Some deep-learning-friendly neuron models, such as the iterative LIF (iLIF) [8] and spiking neural unit (SNU) [9], are customized for scalable SNN configurations. Recently, some neuron models with higher computational complexity have been proposed to improve the performance of SNNs. The Parametric Leaky Integrate-and-Fire (PLIF) spiking neuron [10] allows learning of both synaptic weights and neuronal dynamics through adjustable membrane time constants. The Gated Leaky Integrate-and-Fire (GLIF) spiking neuron [11] incorporates learnable gates to expand integration, decay and reset options, boosting the adaptability of spiking neurons. Inspired by the architecture of long short-term memory (LSTM) units, the LIF neuron combined with after-hyperpolarizing (AHP) currents (LIF–AHP) has been developed to endow SNNs with working-memory capabilities analogous to those of LSTM units [12]. This enhancement enables LIF–AHP neurons to surpass traditional recurrent neural networks in temporal sequencing tasks. Furthermore, the liquid constant neuron is designed to provide a unique solution with an exceptional capacity to capture interventions [13,14]. The inherent causality and richer dynamics of the model are particularly noteworthy. However, these spiking neuron models are only oversimplified abstractions of the sophisticated biological neuron and are hence expected to be enhanced by leveraging more biological insights in depth.

In neuroscience, various fundamental neuron patterns associated with learning and memorizing have been recently uncovered from mammalians [15–28]. At the microscale, the neuron is observed to perform self-inhibiting activity through the autapse from the axon to its soma. The self-inhibiting autapse, regulating spike precision and network activity, forms the biophysical basis for neural memorizing [15]. At the macroscale, the brain is dissected as a heterogeneous network composed of billions of neurons with variant phenotypes [16,17]. At the population-specific heterogeneity level, neural system heterogeneity contributes to the production of stable behaviors in neural networks [18], efficient signal coding [19,20] and robustness to input fluctuations [21]. At the neuron heterogeneity level, variability among neurons enhances the ability to capture rare features of cortical physiology, thereby supporting the fading-memory property [22]. Furthermore, heterogeneity plays a pivotal role in working-memory capacity [23] and memory traces [24] in cortical neurons. At the synaptic heterogeneity level, heterogeneous input synaptic distributions influence organizational activity across different scales in brain dynamics [26]. The importance of adaptive input synaptic properties for temporal structure [27] and stimuli-response tasks [28] has been demonstrated in recent research.

Inspired by these neuroscientific findings, we designed a Heterogeneous spIking Framework with self-Inhibiting neurons (HIFI) for enhanced neural memorizing and learning. HIFI majorly contributes to its heterogeneous configuration that allows variant intra-neuron systems in different neurons. Therefore, unlike traditional SNNs that only need to learn the synaptic weights at the macro network level, HIFI meanwhile requires the learning of neuron-level parameters at the micro neuron level. We hence formulated HIFI learning in a bi-level programming paradigm [29] to optimize these micro- and macro-level parameters in two orthogonal loops. Afterward, each neuron in HIFI learned its unique intra-neuron system for spike processing, behaving analogously to biological neurons in the brain. Overall, HIFI is a more biologically inspired SNN with sophisticated biological inspirations at both neuron and network levels.

We demonstrated the superiority of HIFI from multiple aspects including the fidelity in the biological sense, the performance on AI benchmarks and the potential for scientific exploration. Regarding biological fidelity, the self-inhibiting neuron model in HIFI yielded the most similar artificial spikes and activities of neuronal populations to biological neurons in the context of reproducing neural statistics and supporting neural prostheses compared with previously established models. In AI performance, HIFI outperformed several state-of-the-art SNNs in accuracy, efficiency and latency on eight computer vision and neuromorphic data sets, demonstrating its advances as a practical AI model. For scientific exploration, HIFI can accurately identify the rare cell types of *Sncg, Serpinf1* and *Astro* from single-cell RNA sequences (scRNA-seq). While these rare cells only occupy very minor proportions (e.g. 0.09%), they are key biomarkers for several brain diseases including multiple system atrophy [30] (*Sncg*), glioblastoma [31] (*Serpinf1*) and brain edema [32] (*Astro*). To the best of our knowledge, this is the first time that SNN has been applied and benchmarked to scRNA-seq analysis, in which this efficient and low-latency AI model naturally overcomes the quantity and dimensionality challenges in high-throughput scRNA-seq profiles.

## RESULTS

### HIFI performs heterogeneous learning with biologically inspired self-inhibiting neurons

The advances of HIFI hinge on two propositions: (i) a sophisticated self-inhibiting neuron model and, more importantly, (ii) a brain-like heterogeneous learning framework.

**Figure 1.**
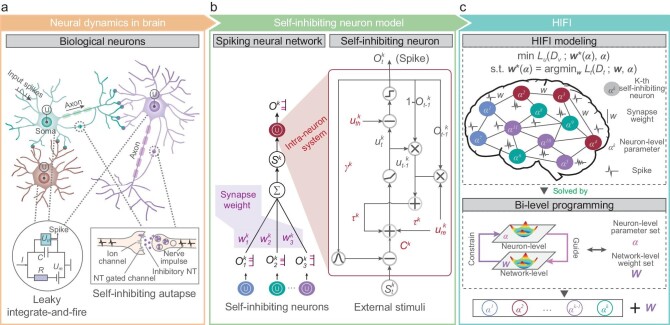
Overview of HIFI. (a) Neuroscientific inspirations for the self-inhibiting neuron model including the leaky integrate-and-fire process and the self-inhibiting autapse. (b) Synapses weights defined at the macro network level and the intra-neuron system defined at the micro neuron level. The learnable parameters group of the *k*th self-inhibiting neuron model is **α**${}^{\boldsymbol{k}} = {\boldsymbol{\ }}[ {{\tau }^k,{\mathrm{\ }}{\gamma }^k,{\mathrm{\ }}{C}^k,{\mathrm{\ }}u_{th}^k,{\mathrm{\ }}u_{re}^k} ]$. (c) HIFI modeling (top). In HIFI, the spiking neural network is composed of heterogeneous self-inhibiting neurons with variant neuron-level parameters **α**${}^k$ and these neurons are connected by synapses with weights ***W***. HIFI learning (bottom). The bi-level HIFI learning is solved by alternating between the neuron-level loop (for ${\boldsymbol{\alpha }}$ updating) and the network-level loop (for ***W***updating).

The proposed self-inhibiting neuron introduces the self-inhibiting autapse [15,33] into the leaky integrate-and-fire process (Fig. 1a). In biology, self-inhibiting autapse considers the influences of past spikes on current incoming inputs, allowing self-control of the memorizing effect in a single neuron [15]. Here, in order to accomplish a neuron-level memorizing effect, we generate the spiking output ${O}^k( t )$ of the *k*th neuron at time point *t* by jointly considering its current stimuli ${S}^k( t )$ and past output ${O}^k( {t - 1} )$ in its intra-neuron system ${F}_{{{\boldsymbol{\alpha }}}^k}( \cdot )$ parameterized by ${{\boldsymbol{\alpha }}}^k \in {\mathbb{R}}^5$, i.e. ${O}^k( t ) = {F}_{{{\boldsymbol{\alpha }}}^k}( {{S}^k( t ),{O}^k( {t - 1} )} )$. The intra-neuron system describes the dynamics of neuronal membrane potential and ion exchange, which are modeled by a set of biophysical equations of a resistor–capacitor (RC) circuit (see Methods, Fig. 1b).

HIFI mimics the heterogeneous nature of brain neural networks by allowing variant neuron-level parameters ${{\boldsymbol{\alpha }}}^k$ on each node (Fig. 1c). Accordingly, unlike traditional SNNs that just learn the network-level synapse weights ${\boldsymbol{W}}$, HIFI also requires learning the neuron-level parameters ${\boldsymbol{\alpha }} = \{ {{{\boldsymbol{\alpha }}}^k} \},k = 1...K$ of *K* composed neurons. Accordingly, we learn these level-wise parameters with a bi-level programming (Fig. 1c):


(1) \begin{eqnarray*}
\begin{array}{@{}l@{}} \mathop {\min }\limits_{\boldsymbol{\alpha }} {\mathcal{L}}_v\left( {{D}_v;{{\boldsymbol{W}}}^*\left( {\boldsymbol{\alpha }} \right),{\boldsymbol{\alpha }}} \right) + \lambda {\mathrm{\Omega }}\left( {{{\boldsymbol{W}}}^*\left( {\boldsymbol{\alpha }} \right),{\boldsymbol{\alpha }}} \right)\\ s.t.\ {{\boldsymbol{W}}}^*\left( {\boldsymbol{\alpha }} \right) = {\mathop{{\rm argmin}}\limits_{\boldsymbol{W}}}{\mathcal{L}}_t\left( {{D}_t;{\boldsymbol{W}},{\boldsymbol{\alpha }}} \right). \end{array}
\end{eqnarray*}

In the constraint, ${\mathcal{L}}_t$ defines the training loss for ${\boldsymbol{W}}$ on the training data set ${D}_t$ with the fixed ${\boldsymbol{\alpha }}$. In the objective, ${\boldsymbol{\alpha }}$ is optimized by trading off the empirical loss ${\mathcal{L}}_v( \cdot )$ and penalization term $\Omega ( \cdot )$ with a hyperparameter $\lambda $. The first empirical loss ${\mathcal{L}}_v( {{D}_v;{{\boldsymbol{W}}}^*( {\boldsymbol{\alpha }} ),{\boldsymbol{\alpha }}} )$ evaluates ${\boldsymbol{\alpha }}$ on the validation data set ${D}_v$ with a learned ${{\boldsymbol{W}}}^*( {\boldsymbol{\alpha }} )$ from the constraint. The penalization term $\Omega ( {{{\boldsymbol{W}}}^*( {\boldsymbol{\alpha }} ),{\boldsymbol{\alpha }}} )$ imposes the Laplacian smoother term to encourage neighboring neurons to share similar neuron-level parameters (Note S1). This penalization term is motivated by our early findings on spatial transcriptomics [16] in which neurons of similar genotypes are spatially grouped on brain tissue. We also propose to use orthogonal sub-training and sub-validation data sets, divided from the whole training data set, in these two levels of optimizations for reducing the chances of over-fitting (Methods, Note S2). The rationale of this bi-level programming framework is also biologically explained by the neuroscientific observations in the brain, in which the synapses can eliminate or form with the evolution of neurons as time passes.

When the constraint of Equation (1) is revisited, the optimization has the same learning objective as those in traditional ANNs and SNNs in which the intra-neuron systems are prefixed and shared homogenously by all neurons (Fig. S1). Therefore, a single objective for weights learning is sufficient for these homogenous neural networks. In HIFI, in addition to these weights ${\boldsymbol{W}}$, the neuron-level parameters ${\boldsymbol{\alpha }}$ should also be learned and hence it requires an extra level of optimization in the objective of Equation (1). After HIFI learning, each neuron covers a unique intra-neuron system, forming a heterogeneous neural network as a brain (Fig. 1c, Methods).

### Self-inhibiting neuron is a high-fidelity neuron model in reproducing neural statistics

**Figure 2.**
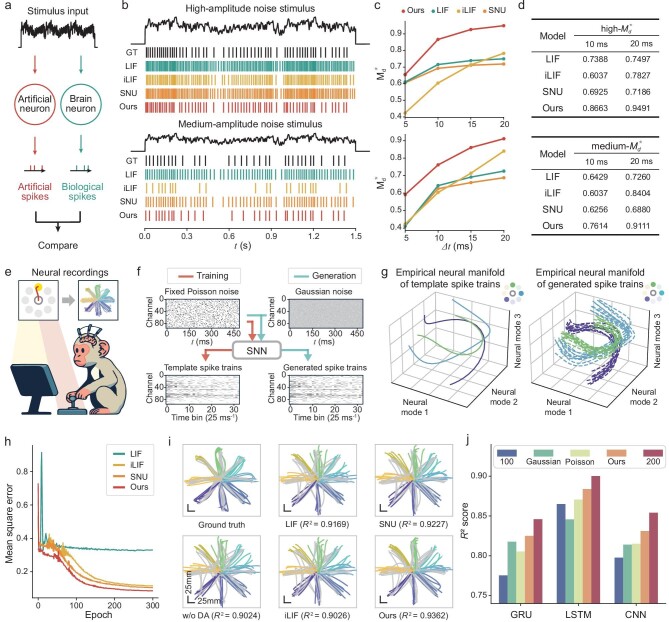
Validating the fidelity of the artificial neuron model on biological spike signals. (a–d) Validation of the capability of reproducing the spiking behavior of neurons in public electrophysiology recordings of Htr3a neuron (specimen ID 474637203). (a) Biological fidelity of a neuron model is quantified by comparing its generated artificial spike trains with the ground-truth biological spike trains in the brain. (b) Raster plots of biological data and optimized models for two types of test data: high-amplitude (upper) and medium-amplitude (bottom) noise current injections. Upper lines represent the injected currents. The first rasters are spikes from recorded biological neurons. Others represent spikes reproduced from the optimized models. Each current injection is 3 s long. (c) Bias-corrected metrics $M_d^*$ for different models at different levels of time-window resolution ${\mathrm{\Delta }}t$, high-amplitude $M_d^*$ (upper) and medium-amplitude $M_d^*$ (bottom). (d) $M_d^*$ of various models at time windows of 10 and 20 ms. (e–j) Assessing the capacity to emulate spiking activity within neuronal populations. (e) Neural recordings recorded by implanted microelectrode array from the monkey executing the center-in-and-out task. (f) SNN is trained to convert fixed Poisson noise into spike trains that resemble biological neural recordings. During the generation phase, additional Gaussian noise is introduced into the input to increase variability. (g) Empirical 3D neural manifold derived from the template spike trains (three out of nine reaching conditions, left) and the corresponding generated spike trains across 10 trials (right). (h) Learning curves for networks with varying spiking neuron models in reproducing biological spiking activities. (i) Decoded cursor trajectories and corresponding *R* ^2^ similarity scores, using the GRU decoder with augmented neural recordings generated from different SNNs. w/o DA: without data augmentation. (j) *R* ^2^ scores with or without data augmentation are compared on various trained decoders and the quantities of original neural recordings for training are varied.

To investigate the fidelity of the self-inhibitory neuron, we applied it to reproduce neural statistics obtained from electrophysiological recordings of the Allen Mouse Brain (Fig. 2a–d). Moreover, we used self-inhibiting neurons to construct an SNN for replicating biological activation patterns of neuronal populations from electrophysiological recordings of rhesus macaques [34]. We then evaluated its efficacy through downstream brain–computer interface (BCI) decoders (Fig. 2e–j).

Regarding electrophysiological data of the mouse brain, we utilized publicly available intracellular electrophysiological recordings from the Allen Cell Type Database [35] to assess the ability of a self-inhibiting neuron to replicate the neural statistics of biological neurons (Fig. 2a and Note S3). Owing to the fact that noise stimulus is more relevant to realistic current injections of biological neurons [36], we evaluated the performance of various neuron models under two types of noise stimuli (Fig. 2b) after training using the long-square stimulus (Fig. S2). We compared the performance of a self-inhibiting neuron (ours) against other artificial neuron models including LIF [6], iLIF [37] and SNU [9]. The resulting raster plots for spikes (Fig. 2b) demonstrated that our self-inhibiting neuron more accurately replicated the spike timings of biological neurons compared with other similar models. To quantitatively measure the performance of neuron models in reproducing neuronal spiking behavior, we used the normalized, bias-corrected metric $M_d^*$ (Note S3), which is commonly employed to assess the similarity between sets of spike trains in electrophysiological recordings [38]. We calculated the $M_d^*$ of neuron models across different time-window resolutions (Fig. 2c). The results revealed that the self-inhibiting neuron model could reproduce maximal 87% and 95% of the spike times at resolutions of 10 and 20 ms, respectively, when tested on an *in vivo*-like stimulus for biological neurons (Fig. 2d).

Moreover, we noticed that our neuron model improved LIF by only introducing the self-inhibiting link. With this unique design, in tasks for reproducing biological neural statistics, our neuron model outperformed LIF upon both visual inspection (Fig. 2b) and quantitative study (Fig. 2c and d). Such a comparison served as an ablation study to partially verify the contributions of the self-inhibiting links in our model design.

Having assessed the biological fidelity at the individual neuron level, we further extended our analysis to the neuronal population level. The distributed and coordinated activity of neuronal populations, which can be measured through implantable intracortical microelectrodes, forms the basis of mammalian behavior [39] and is frequently used in neuroprostheses and neuroprosthetics applications [40]. Due to the success achieved by deep learning across various domains, it also has been introduced to decode neural activity, yielding impressive results [41]. However, data instability and scarcity that derive from the variability of biological samples and the high cost of biological experiments hinder the advancement of data-driven deep-learning models in BCI decoding. To alleviate the problem, we employed a bio-interpretable SNN framework with self-inhibiting neurons to generate biologically plausible activation patterns of neuronal populations by following recent neurological data augmentation methods [42,43].

**Figure 3.**
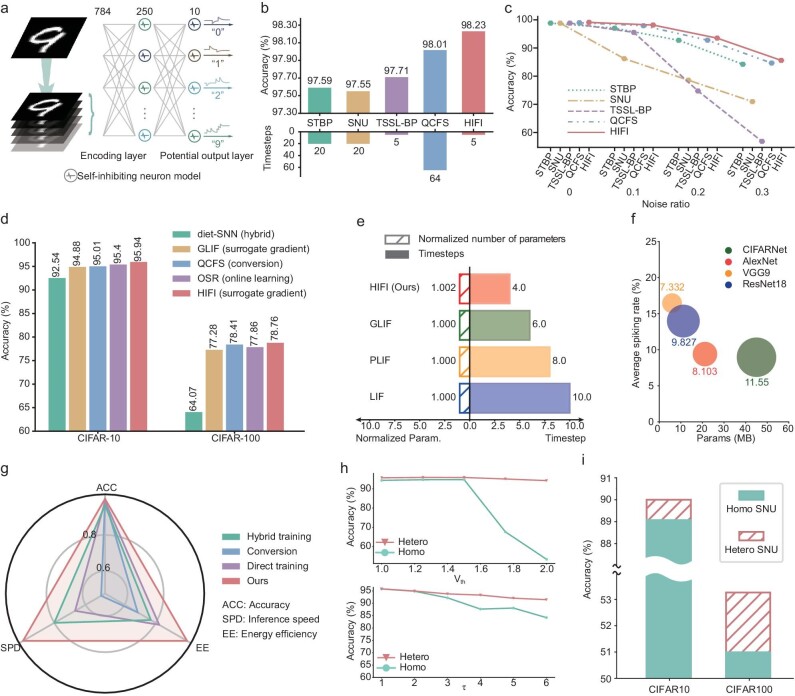
Evaluation of HIFI on computer vision data sets. (a) Fully connected architecture of HIFI for handwritten digit recognition. (b) Classification accuracy and latency (evaluated as time steps) comparisons of different SNNs on MNIST. (c) Noise robustness evaluation of different SNNs on MNIST. (d) Classification accuracy comparisons of different SNNs on CIFAR10 and CIFAR100. (e) Relative parameter and latency comparisons of SNNs with different spiking neuron models. (f) Energy comparisons of HIFI over the analog-valued CNNs on CIFAR10. (g) Radar plots compare the performance of different SNNs in three aspects on CIFAR10 (see Methods). (h) Classification accuracy comparisons for HIFI trained by heterogeneous learning or homogeneous learning (only the synaptic weights are learned) on CIFAR10 in different threshold (${V}_{{\mathrm{th}}}$) initializations and membrane decay constant ($\tau $) initializations. Hetero: heterogeneous learning; Homo: homogeneous learning. (i) Classification accuracy comparisons for SNU-based SNNs optimized with their original algorithm and with our heterogeneous learning on CIFAR10 and CIFAR10. Homo SNU.: original algorithm.

Specifically, SNNs were employed to simulate neural recordings that closely resembled those obtained from the motor cortex of a monkey performing the center-out-and-back task, in which the monkey manipulates a device to move a 2D cursor on a screen from the center point to the surrounding point or vice versa (Fig. 2e and Note S3). Cheap noise signals were fed into the SNN to generate simulated neural recordings (Fig. 3f and Note S3). To evaluate the biological fidelity of the generated activation spike trains, we introduced neural modes metrics [44] (Fig. 2g), mean squared error (MSE) metrics (Fig. 2h) and cursor velocity decoder metrics to compare SNNs that featured different spiking neurons (Fig. 2i and j, Note S3 and Fig. S3).

In neuroscience, neural modes, which represent distinct activation patterns of neuronal populations, can be derived empirically from recorded neural activation patterns using dimensionality reduction techniques [44]. To evaluate the similarity between the generated spike trains and the original template data, we projected the spiking patterns onto a low-dimensional manifold using principal component analysis and visualized the neural modes (Fig. 2g). The results demonstrate that our SNN emulated the activation patterns of the template ground truth (Note S3) at the manifold level. Additionally, the MSE curves indicate that our self-inhibiting neuron model outperforms other comparable models in learning performance (Fig. 2h). Collectively, these results indicate that SNNs equipped with self-inhibiting neurons have a superior capacity to generate stable and diverse data with biological dynamics.

Subsequently, we trained a BCI decoder of gated recurrent unit (GRU), which can extract cursor velocity from neural recordings, with or without the augmented data to further evaluate the biological fidelity of the generated activation spike trains (Note S3). To assess the effectiveness of the augmented data, we analysed the cursor trajectories and the corresponding *R* ^2^ scores of the decoding results (Fig. 2I and Note S3), demonstrating qualitatively and quantitatively that SNNs with self-inhibiting neurons can better reproduce the neural statistics of neuronal populations. Furthermore, SNNs with self-inhibiting neurons were incorporated into three prevalent decoder architectures including GRU, LSTM and CNN, respectively (Fig. 2j and Note S3). All three decoders exhibited substantial performance improvements when trained with our generated neural recordings, showing the generality of our model as a biologically plausible argumentation tool. Moreover, by adapting the number of original biological neural recordings (*N*) in the training data (Fig. 2j), we observed that our reproduced neural recordings could significantly enhance the performance of data-driven deep-learning decoders, especially in data-scarce scenarios. To further validate the effectiveness of our generated neural recordings, we compared the performance of decoders trained on original data or data that were augmented using various methods (Fig. S4). The results demonstrate that our generated neural recordings consistently achieve higher *R*² scores across all decoders compared with those augmented with Gaussian and Poisson methods, underscoring the superior effectiveness. The observed performance differences when training decoders with varying numbers of original biological neural recordings (*N* = 100, 200) highlight the inherent variability in biological data. Also, the higher *R*² scores suggest that our neural recordings effectively mitigate this variability by enhancing the diversity and robustness of the training data set, thus accurately representing biological characteristics and fidelity.

In conclusion, the proposed self-inhibiting neuron exhibits high capacity in reproducing the neural statistics of brain neurons and is hence an ideal building block for SNN configurations.

### HIFI achieves sound performance on computer vision tasks

With the proposed neuron model as the basic building block , we constructed HIFIs and evaluated their image classification performance on the MNIST [45], CIFAR10 [46], CIFAR100 [46] and ImageNet [47] data sets compared with other state-of-the-art SNNs.

In MNIST, we directly followed the same network architecture design in SNU [9] to configure HIFI with three fully connected layers (784–250–10) (Fig. 3a). We compared HIFI with some representative backpropagation and conversion SNNs including STBP [37], SNU [9], TSSL-BP [6] and optimal conversion [48] (QCFS). HIFI showed the highest accuracy (98.23%) and the lowest latency (5 time steps) among all compared SNNs (Fig. 3b and Table 1). While the second-most accurate SNN (QCFS) achieved comparable accuracy, its latency was 13 times higher than that of HIFI. Furthermore, we considered adding different types of noises to verify the robustness of different AI models with noise perturbations including salt-and-pepper noise, zero-mean Gaussian noise and speckle noise. The noise was not involved in training but was added for the testing samples. HIFI was robust against noises of different types and levels (Fig. 3c and Fig. S5).

**Table 1.** Comparison of HIFI with state-of-the-art SNNs.

Data set	Method	Architecture	Time step	Accuracy (%)
CIFAR10	PLIF [10] *^’ICCV2021^*	PLIF Net	6	94.25
	Dspike [49] ^’NIPS2021^	ResNet-18	6	93.50
	DSR [50] *^’CVPR2022^*	PreAcr-ResNet-18	20	95.40
	GLIF [11] *^’NIPS2022^*	ResNet-18	6	94.88
	TET [51] *^’^ ^ICLR 2022^*	ResNet-19	6	94.50
	QCFS [48] *^’ICLR2022^*	ResNet-18	8	94.82
	SLTT [52] *^’ICCV2023^*	ResNet-18	6	94.59
	Diet-SNN [53] *^’TNNLS2023^*	ResNet-20	10	92.54
	OSR [54] *^’ICLR2024^*	ResNet-19	4	95.20
	TAB [55] *^’ICLR2024^*	ResNet-19	6	94.81
	**HIFI (Ours)**	**ResNet-18**	**2**	**95.67**
			**4**	**95.94**
			**6**	**95.98**
CIFAR100	Dspike [49] ^’NIPS2021^	ResNet-18	6	74.24
	DSR [50] *^’CVPR2022^*	PreAcr-ResNet-18	20	78.50
	GLIF [11] *^’NIPS2022^*	ResNet-18	6	77.28
	TET [51] *^’^ ^ICLR2022^*	ResNet-19	6	74.72
	QCFS [48] *^’ICLR2022^*	ResNet-18	8	78.48
	SLTT [52] *^’ICCV2023^*	ResNet-18	6	74.67
	Diet-SNN [53] *^’TNNLS2023^*	ResNet-20	5	64.07
	OSR [54] *^’ICLR2024^*	ResNet-19	4	77.86
	TAB [55] *^’ICLR2024^*	ResNet-19	6	76.82
	**HIFI (Ours)**	**ResNet-18**	**2**	**77.80**
			**4**	**78.76**
			**6**	**79.32**
ImageNet	Dspike [49] ^’NIPS2021^	ResNet-34	6	68.19
	DSR [50] *^’CVPR2022^*	PreAcr-ResNet-18	50	67.74
	GLIF [11] *^’NIPS2022^*	ResNet-34	4	67.52
	TET [51] *^’ICLR20222^*	SEW-ResNet-34	4	68.00
	QCFS [48] *^’ICLR2022^*	VGG-16	32	67.73
	SLTT [52] *^’ICCV2023^*	NF-ResNet-34	6	66.19
	Diet-SNN [53] *^’TNNLS2023^*	VGG-16	5	69.00
	OSR [54] *^’ICLR2024^*	NF-ResNet-34	4	67.54
	TAB [55] *^’ICLR2024^*	ResNet-34	4	67.78
	**HIFI (Ours)**	**SEW-ResNet-34**	**4**	**69.11**

Underlined digits denote the best accuracy among baseline SNNs.

In CIFAR10 and CIFAR100, HIFI was compared with state-of-the-art SNNs. HIFI achieved the best performance in accuracy and latency with the relative number of parameters, showing its great potential in computer vision tasks (Fig. 3d and e, and Table 1). Next, we analysed the operational complexity of different spiking neurons (Table S1) and compared SNNs with these neurons on a specific architecture (Fig. S6). The results indicate that the operational complexity among various spiking neurons shows only slight differences and our self-inhibiting neuron minimally increase operational complexity during inference. Moreover, we applied HIFI to the more challenging ImageNet data set to evaluate its performance. HIFI achieved superior accuracy and latency compared with different SNNs (Table 1). After energy consumption was evaluated by using floating-point representation on CMOS technology (Note S4), HIFI exhibited substantial energy efficiency improvements across various data sets compared with the corresponding ANNs (Fig. 3f and Fig. S7a). For example, HIFI achieved a 9.83-fold enhancement in energy efficiency with a time step of 4 (Fig. 3f) and a maximum improvement of 17.83-fold with a time step of 2 on CIFAR10 (Fig. S7a). Additionally, as fixed-point representation is commonly utilized to evaluate the energy consumption of SNNs (Note S4), we also compared the energy consumption of HIFI by using fixed-point operations with ANNs (Fig. S7b). HIFI achieved maximally 85.73-fold reductions in energy consumption compared with ANNs. Given that neuromorphic devices are specifically designed to optimize computational efficiency for SNNs, we further assessed the energy consumption of HIFI on neuromorphic platforms (e.g. TrueNorth and SpiNNaker) and compared it to that of SNNs (Note S4 and Table S2). HIFI consistently achieved superior energy efficiency compared with other state-of-the-art SNNs. Overall, HIFI performs best among different SNN learning methods in accuracy, inference speed and energy efficiency (Fig. 3g), showing HIFI to be an efficient model for neuromorphic computing.

We further conducted an ablation study on CIFAR10 to understand the contributions of heterogeneous learning in HIFI. We compared the performance of HIFIs trained by heterogeneous learning or homogeneous learning under different initializations on two important hyperparameters of SNN (Fig. 3h). Our findings indicated that heterogeneous learning greatly improved the accuracy under different initializations, even with inferior hyperparameter configurations, compared with homogeneous learning. Additionally, we used our heterogeneous learning to train an SNU-based SNN on CIFAR10 and CIFAR100. The heterogeneous learning approach also improved the accuracy performance of the SNU (Fig. 3i). This study suggests that heterogeneous learning can significantly bolster the performance of models, enabling the efficient training of SNNs to surpass traditional approaches that rely on costly and exhaustive searches for optimal hyperparameters.

Moreover, we trained HIFIs by allowing different degrees (1, 2 and 5) of freedoms in the neuron model and compared the performance of the simplified HIFIs with different state-of-the-art (SOTA) SNNs (Table S3). The results showed that the performance of HIFI was only slightly affected by reducing the degrees of freedom (Table S3). Meanwhile, even when the degree was reduced to 1, the performance of HIFI still outperformed the SOTA SNNs.

Notably, recent work [56] has proposed learnable inter-layer feedback connections to suppress the membrane potential of spiking neurons in the current layer, thereby improving the performance of SNNs in machine learning. To thoroughly evaluate the effectiveness of our proposed self-inhibiting spiking neurons, we compared its performance by using the inter-layer feedback method (Fig. S8). First, we compared SNNs with self-inhibiting neurons and inter-layer feedback connections on MNIST and CIFAR-10, in which our model achieved superior accuracy (Fig. S8b and c). Additionally, in order to evaluate the capability for processing dynamic sequences, we evaluated SNNs on music prediction tasks (Fig. S8d). Our model more accurately predicted the probabilities of consecutive music notes, demonstrating an enhanced ability to process temporal sequences and adapt to changing inputs (Fig. S8e). Due to the additional learnable parameters, SNNs with inter-layer feedback connections require more training time and are less efficient than our model (Fig. S8e). These results collectively highlight the significant advantages of self-inhibiting spiking neurons in terms of accuracy, dynamic temporal processing and training efficiency compared with SNNs with learnable inter-layer feedback connections.

In conclusion, HIFI is an accurate, energy-efficient and low-latency SNN in addressing computer vision tasks.

### HIFI outperforms other SNNs and shows good generalization ability on neuromorphic data

**Figure 4.**
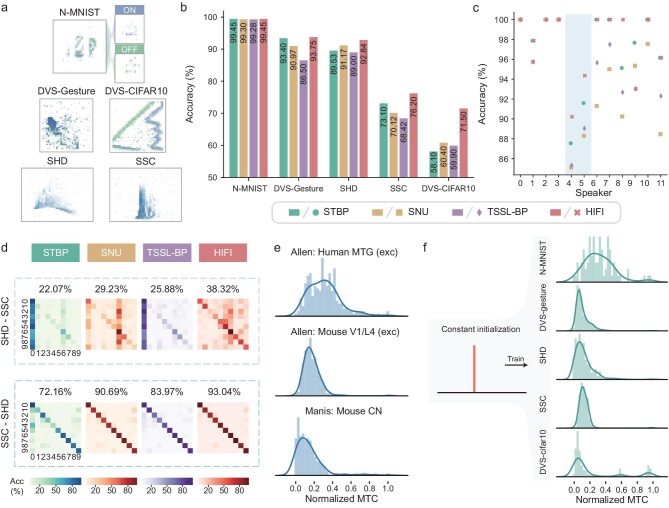
Evaluation of HIFI on neuromorphic data sets. (a) Visualizations of neuromorphic data sets. N-MNIST with ‘on’ and ‘off’ spikes (top). DVS128-Gesture with 11 different actions (middle). DVS-CIFAR10 converted from CIFAR-10 with 10 different classes, SHD with English and German recordings and SSC with English recordings (bottom). (b) Classification accuracy for different SNNs on four neuromorphic data sets. (c) Classification accuracy on the test set of SHD for different SNNs across 12 speakers, where only Speaker 4 and Speaker 5 are involved in the test data set. (d) Confusion matrices and classification accuracy of different SNNs. SHD–SSC represents the scenario of training on the SHD but testing on the SSC and vice versa. The classification accuracy of each model is labeled above the corresponding confusion matrix. (e) Experimentally observed distributions of membrane time constants (MTCs) for human middle temporal gyrus [35,57,58] (spiny cell, 236 cells), mouse V1 layer 4 [35,57,58] (spiny cell, 164 cells) and mouse cochlear nucleus [59] (multi cell types, 172 cells). (f) Membrane time-constant distributions before (left) and after (right) training for each data set.

With the superior performance achieved in standard static computer vision tasks, we further evaluated HIFI on neuromorphic data sets for demonstrating its ability to process spatio-temporal sequences. The neuromorphic data sets are specifically designed for SNNs that contain spike-based data recorded by bio-inspired vision or audio devices, e.g. asynchronous vision sensors [60–62] and the artificial inner ear [63]. Here, we evaluated HIFI on five neuromorphic data sets including three neuromorphic video data sets (N-MNIST [60], DVS128-Gesture [61] and DVS-CIFAR10 [62]) and two neuromorphic audio data sets [63] (SHD and SSC) (Fig. 4a). HIFI was compared with three SNNs including STBP, SNU and TSSL-BP with a shallow convolutional network architecture (Note S5).

We first verified the performance of HIFI on different data sets. With the same convolutional architecture, HIFI outperformed other baselines on all tasks with higher accuracy (maximally 8% improvement) and lower latency (5 time steps, maximally 4-fold reduction) (Fig. 4b and Table S4). We then evaluated the generalization ability of HIFI across speakers (Fig. 4c) and data sets (Fig. 4d). For generalization across different speakers (Fig. 4c), HIFI showed the best performance on two held-out speakers (Speakers 4 and 5). For generalization across different data sets, we trained a model on one data set (e.g. SHD) but tested on the other (e.g. SSC) and vice versa. HIFI showed the best cross-data set generalization performance compared with other SNNs (Fig. 4d). From the confusion matrices in the SHD–SSC tests, STBP classified almost all digits to the digit 0. Such misclassification phenomena were also observed from the confusion matrices of other competing SNNs. HIFI showed the strongest signals in the diagonal of the confusion matrices and hence gained the best overall classification accuracy in this cross-data set test.

We further compared the distributions of the trained neurobiological parameters with the experimentally observed parameters of mammalian neurons [35,59]. The compared neurobiological parameters included the resting membrane potentials (RMPs) and the membrane time constants (MTCs). We visualized and compared the distributions of MTCs (Fig. 4e and f) and RMPs (Fig. S9), recorded in real brain neurons and in HIFI's neurons that were learned on different neuromorphic data sets. The parameters of neurons trained in HIFI and the experimentally observed parameters share very similar distributions. The results support that the parameters of heterogeneously learned neurons are partially neurobiologically meaningful.

In conclusion, HIFI shows superior performance on all neuromorphic data sets, better generalization ability across speakers and data sets on audio classifications, and potential to handle future complex tasks.

### HIFI is capable of identifying rare cell types from scRNA-seq data

With HIFI having been proven as a useful machine-learning tool in classifying images and processing spatio-temporal sequences, we further evaluated HIFI on scRNA-seq data to demonstrate its ability at performing more general machine-learning tasks. scRNA-seq enables a high-resolution atlas of individual cells, which rapidly advances our understanding of the cellular heterogeneity of complex tissues [64]. The identifications of cell types from scRNA-seq data sets are critical for facilitating many down-streaming biological discoveries. Since the quantity and dimensionality of scRNA-seq are both large, an accurate, efficient and low-latency computational framework is highly desired in the field [65]. Here, we consider using SNNs for conducting cell type identification. To the best of our knowledge, this is the first time that SNNs have been benchmarked on this prevalent bioinformatics task.

**Figure 5.**
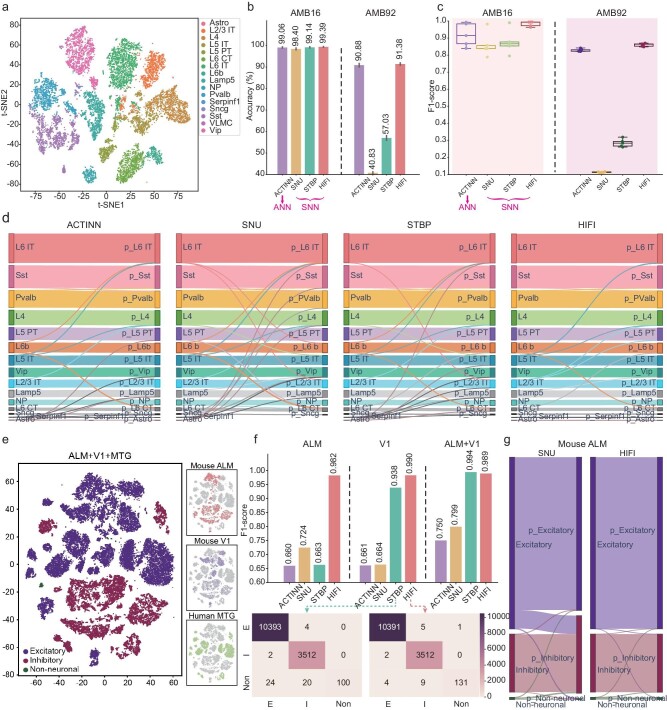
Evaluation of HIFI for cell type identification on four RNA-seq data sets. (a–d) Comparisons of SNNs on the Allen Mouse Brain Atlas with two levels of annotations, 16 (AMB16) and 92 (AMB92), after eliminating minor cell types. (a) tSNE visualizations of scRNA-seq from AMB16. (b) Average prediction accuracy of 5-fold data for different neural networks on AMB16 (left) and AMB92 (right). (c) Box-plot visualization of F1-scores for AMB16 (left) and AMB92 (right). Box plot: median (center line), interquartile range (box) and data range (whiskers). (d) Sankey plots of different neural networks on AMB16. Sankey plot: cell numbers of each ground-truth cell type (left nodes), cell flows from ground truth to prediction (middle link), cell numbers of each predicted cell type (right nodes). (e–g) Comparison of SNNs on three different brain data sets across species. (e) tSNE visualizations of the combined scRNA-seq data. (f) Prediction performance across different brain data sets. Bar plots show the F1-scores of different neural networks using the paired ALM-MTG (left), V1-MTG (middle) and ALM + V1-MTG (right) data sets. Confusion matrices show the detailed prediction results of STBP and HIFI on V1-MTG. E: excitatory, I: inhibitory, Non: non-neuronal. (g) Sankey plots of SNU and HIFI on ALM-MTG.

First, we evaluated the performance of HIFI in cell type identification, especially in identifying rare cell types, on the Allen Mouse Brain [32] data set with 16 annotations (AMB16, Fig. 5a) and 92 deep annotations (AMB92, Fig. S10), respectively. We compared HIFI with ACTINN [65] (a specialized ANN designed for cell type identification) and two SNNs, i.e. STBP and SNU. On AMB16, HIFI achieved the best classification accuracy (Fig. 5b, left) and F1-score (Fig. 5c, left) compared with the other models. The higher F1-score of HIFI indicated its better sensitivity in discovering rare cell types. In biology, these rare cells are conventionally major driving factors for some complex phenomena, e.g. the formation of cancer [31]. To be specific, HIFI successfully identified the rare cell types of *Sncg, Serpinf1* and *Astro*, which were all neglected by other approaches (Fig. 5d and Fig. S11). These rare cell types are very informative biomarkers for severe diseases. For example, the *Sncg* cell type has been reported in neurotic pathology of multiple system atrophy [30], the *Serpinf1* is downregulated in primary malignant brain tumor [31] (i.e. glioblastoma) and the *Astro* deletion reduces the brain edema [32]. On AMB92, the performance of two competing SNNs (SNU and STBP) dropped sharply, while HIFI still maintained the highest accuracy and F1-score (Fig. 5b, right, Fig. 5c, right, and Fig. S12). Such results indicate that HIFI can precisely identify subpopulations of cells with very deep annotation levels.

In biology, the cross-species tests help to uncover the evolutionary specializations and commonalities across species. Here, we evaluated the performance of HIFI across different species on three brain data sets including Mouse ALM [32], Mouse V1 [32] and Human MTG [66]. While these data sets were sequenced from different brain regions (ALM vs. V1) or from different species (mouse vs. human), they were embedded to very nearby locations in the tSNE space (Fig. 5e). We then paired three ‘train-test’ data sets, i.e. ALM-MTG, V1-MTG and ALM + V1-MTG. HIFI achieved the best F1-scores on all three paired ‘train-test’ data sets (Fig. 5f and Note S6), showing the good generalization ability and robustness of HIFI at extracting common genetic features across species. Furthermore, both common and rare cell types (non-neuronal) across species were precisely predicted using HIFI but other SNNs and ACTINN failed (Fig. 5g and Fig. S13). The comparison results show that HIFI can better remove the biological differences between species across data sets of different species and provide a new tool for analysing the correlations across different species.

In summary, HIFI can accurately identify rare yet informative biomarkers and better represent specializations and commonalities across species.

## DISCUSSION

As broadly recognized brain-inspired AI models, ANNs have achieved very promising accuracy on many benchmarks. However, the sound accuracy of ANNs is conventionally obtained at the cost of expensive energy consumption and slow inference speed. Alternatively, SNNs alleviate the heavy computational burden of ANNs by adopting a more biologically inspired framework for spike computations. Here, we consider further improving the accuracy, efficiency and speed of SNNs by leveraging recent neuroscientific findings in both neuron and network design.

For neuron design, inspired by the self-inhibiting autapse, we proposed a self-inhibiting neuron model by granting memorizing ability into each neuron. This neuron model provides a new perspective for modeling and understanding the complicated neural dynamics of mammalian brain neurons. To confirm its biological fidelity, we used the self-inhibiting neuron model to reproduce neural statistics at the single neuron level and neuronal population level. The proposed model outperformed other spiking neuron models (LIF, iLIF and SNU) in reproducing the spiking activity of biological brain neurons.

For network design, inspired by the heterogeneity of the brain, we proposed a HIFI SNN configured by heterogeneous self-inhibiting neurons. Bi-level programming was formulated to successfully conquer the variable hierarchy difficulties in HIFI learning. On image and neuromorphic data sets, HIFI achieved as high accuracy as conventional ANNs and exhibited great advantages in inference speed and energy consumption. When compared with other SNNs, HIFI could beat all compared baselines in terms of accuracy, efficiency and latency on all data sets.

The high efficiency and low latency of HIFI further motivate us to use it for analysing scRNA-seq on a large scale with high dimensionality. In cell type identification, HIFI outperformed all compared SNNs, as well as the ANN that was specifically designed for this task. More importantly, HIFI accurately identified the rare cell types of *Sncg, Serpinf1* and *Astro* while other SNNs and ANN failed. These discovered cell types are very informative biomarkers for several brain diseases including multiple system atrophy (*Sncg*), glioblastoma (*Serpinf1*) and brain edema (*Astro*). In the scenario of the cross-species test, the robustness and generalization of HIFI were again witnessed in correctly extracting common cross-species genetic features from mouse and human brains.

In conclusion, this interdisciplinary work contributes to biologically inspired AI. HIFI offers an accurate, efficient and low-latency learning framework for general machine-learning tasks.

## CHALLENGES AND OUTLOOK

The HIFI model, while presenting significant advancements, faces several limitations and challenges that need to be addressed in future research. A primary issue with the HIFI model is its computational complexity, as the advanced algorithms demand substantial processing power and memory. This requirement limits the applicability of the model in resource-constrained environments, such as mobile devices or embedded systems. Although the integration of the HIFI model with neuromorphic devices holds promise for reducing energy consumption and enhancing data memory efficiency, it also introduces significant challenges related to compatibility and deployment. For real-time applications, the integration of HIFI with brain–computer interfaces presents additional challenges, particularly in ensuring that the model processes data without latency issues. Additionally, the effectiveness of the model is dependent upon the availability and quality of the data, and insufficient or low-quality data can adversely affect accuracy and reliability. It is crucial that these limitations and challenges are addressed to enable broader adoption and application of the HIFI model across various fields.

In response to these challenges, our future work will focus on several key areas to enhance the robustness, efficiency and adaptability of the HIFI model. Firstly, we plan to explore the integration of the HIFI model with neuromorphic devices. These devices offer significant advantages in terms of processing and storage efficiency, which can further enhance the performance of the HIFI model. Digital circuits are universally employed for implementing neuron compute units in neuromorphic chips such as TrueNorth, Loihi and SpiNNaker. Therefore, we will analyse the feasibility of implementing our spiking neuron model on neuromorphic chips using digital circuits. Our model, described by five core equations (see Methods), includes an additional self-inhibition process compared with the traditional LIF model. For the input weighted summation (${S}^k( t )$), multipliers and adders in parallel can efficiently compute the weighted sums, leveraging the maturity of modern digital circuits. The self-inhibition process (${I}^k( t )$) can be achieved by using simple subtractors and logic circuits. The membrane potential update (${u}^k( t )$) involves multipliers, adders and a lookup table (LUT) for the function *f*, ensuring high efficiency. The spike emission (${O}^k( t )$) is implemented using comparators to check whether the membrane potential ${u}^k( t )$ exceeds the threshold $u_{th}^k$. The membrane potential reset (${u}^k( t )$) is managed by multipliers and adders, controlled by using simple logic circuits. Specifically, the heterogeneity, which represents the unique parameters of each neuron (${\mathrm{\ }}[ {{\tau }^k,{\mathrm{\ }}{\gamma }^k,{\mathrm{\ }}{C}^k,{\mathrm{\ }}u_{th}^k,{\mathrm{\ }}u_{re}^k} ]$), can be stored in dedicated SRAM memory. These parameters can be dynamically loaded from memory, allowing fine-tuning and customization at the individual neuron level. Digital circuits are well suited to these operations due to their high precision, programmability and ease of integration into large-scale networks. The programmability and flexibility of digital circuits, especially with field-programmable gate array based designs, facilitate rapid iteration and optimization. Overall, the implementation of our spiking neuron model that uses digital circuits on neuromorphic chips is highly feasible.

Additionally, we intend to investigate the combination of the HIFI model with brain–computer interfaces (BCIs). The development of BCI technology offers new possibilities for real-time data processing and response. Integration of the HIFI model with BCIs could open up new application areas in neuroscience and clinical applications. The efficient data-processing capabilities of HIFI could enable rapid analysis and real-time feedback of brain signals, thereby improving the response speed and accuracy of BCI systems. Additionally, to address the data dependence issue, we will focus on developing advanced data augmentation and preprocessing techniques to improve the quality and reliability of the input data, ensuring consistent performance of the HIFI model across diverse data sets. Through these explorations, we aim to further expand the application range of the HIFI model and leverage its potential in multiple fields.

## METHODS

### Self-inhibiting neuron model

The underlying dynamics of our self-inhibiting neuron model can be interpreted by the following equations, step-by-step:


(2) \begin{eqnarray*}
{S}^k\left( t \right) = \mathop \sum \limits_{i \in N\left( k \right)} {w}_{ik}{O}^i\left( {t - 1} \right)
\end{eqnarray*}

(3) \begin{eqnarray*}
{I}^k\left( t \right) = {S}^k\left( t \right) - {\gamma }^k \cdot {O}^k\left( {t - 1} \right)
\end{eqnarray*}

(4) \begin{eqnarray*}
{u}^k\left( t \right) &=& f[ \left( {1 - {\tau }^k} \right){u}^k\left( {t - 1} \right)\\
&& +\, {\tau }^ku_{re}^k + {C}^k{I}^k\left( t \right) ]
\end{eqnarray*}

(5) \begin{eqnarray*}
{O}^k\left( t \right) = \Theta \left( {{u}^k\left( t \right) - u_{th}^k} \right)
\end{eqnarray*}

(6) \begin{eqnarray*}
{u}^k\left( t \right) = \left( {1 - {O}^k\left( t \right)} \right){u}^k\left( t \right) + {O}^k\left( t \right)u_{re}^k
\end{eqnarray*}

Equation (2) defines the external stimuli ${S}^k( t )$ of the *k*th neuron from its neighbors at step *t*, where $N( k )$ denotes all connected neurons of the *k*th neuron, ${w}_{ik}\ $defines the synapse weight from neuron *i* to *k* and ${O}^i( {t - 1} )$ is the last-step output from neuron *i*.

Equation (3) models the self-inhibiting phenomenon that the final incoming input ${I}^k( t )$ to the *k*th neuron simultaneously considers the external stimuli ${S}^k( t )$ and its last-step output ${O}^k( {t - 1} )$. ${\gamma }^k$ is the neuron-wise adaptive parameter.

Equation (4) describes the membrane potential ${u}^k( t )$ of the *k*th neuron at step *t*, where $f( \cdot )$ is the Leaky-ReLU function, and ${\tau }^k$, ${C}^k$ and $u_{re}^k$ are the membrane decay, capacitance and resting potential, respectively.

Equation (5) models the output of the *k*th neuron at step *t* by comparing the current membrane potential ${u}^k( t )$ with the inherent threshold potential $u_{th}^k$ of the neuron in a Heaviside step function ${\mathrm{\Theta }}( x )$, which will generate a spike when $x > 0$.

Equation (6) describes the membrane potential with or without firing. Once firing, ${O}^k( t ) = 1$ and it resets the membrane potential to the resting potential $u_{re}^k$. Otherwise, it maintains the current potential.

All biophysical variables of our self-inhibiting neuron model are learnable and defined in the parameters ${{\boldsymbol{\alpha }}}^k = \ [ {{\tau }^k,{\mathrm{\ }}{\gamma }^k,{\mathrm{\ }}{C}^k,{\mathrm{\ }}u_{th}^k,{\mathrm{\ }}u_{re}^k} ]$, that all vary in a neuron-wise way.

### HIFI learning

HIFI learning can be performed by iteratively alternating between the network-level and neuron-level loops (see Note S2 for more details about the bi-level optimization). The network-level loop adopts a surrogate gradient [9] to update the synapse weight via ${\boldsymbol{W}} \leftarrow {\boldsymbol{W}} - {\xi }_1{\nabla }_{\boldsymbol{W}}{\mathcal{L}}_t( {{D}_t;{\boldsymbol{W}},{\boldsymbol{\alpha }}} )$, where ${\xi }_1$ is the learning rate and${\mathrm{\ }}{\boldsymbol{\alpha }}$ is fixed from the neuron-level loop. The neuron-level loop updates neuron-level parameters via ${\boldsymbol{\alpha }} \leftarrow {\boldsymbol{\alpha }} - {\xi }_2{\nabla }_{\boldsymbol{\alpha }}{\mathcal{L}}_u( {{D}_v;{{\boldsymbol{W}}}^*( {\boldsymbol{\alpha }} ),{\boldsymbol{\alpha }}} )$, where ${\mathcal{L}}_u\ $denotes the whole neuron-level objective function and ${\xi }_2$ is the learning rate in the neuron-level loop. The calculation of ${\nabla }_{\boldsymbol{\alpha }}{\mathcal{L}}_u( {{D}_v;{{\boldsymbol{W}}}^*( {\boldsymbol{\alpha }} ),{\boldsymbol{\alpha }}} )$ is not easy because the optimal value of ${{\boldsymbol{W}}}^{\boldsymbol{*}}( {\boldsymbol{\alpha }} )$ is also a function of ${\boldsymbol{\alpha }}$. Here, we followed common practices [67] in existing bi-level optimization works to approximate ${{\boldsymbol{W}}}^{\boldsymbol{*}}( {\boldsymbol{\alpha }} )$ with its one-step update and then derived the gradient of ${\mathcal{L}}_u( \cdot )$ with respect to ${\boldsymbol{\alpha }}$ via the chain rule:


(7) \begin{eqnarray*}
&&{\nabla }_{\boldsymbol{\alpha }}{\mathcal{L}}_u\left( {{{\boldsymbol{W}}}^{\boldsymbol{*}}\left( {\boldsymbol{\alpha }} \right),{\boldsymbol{\alpha }}} \right)\\
&&\quad \approx {\nabla }_{\boldsymbol{\alpha }}{\mathcal{L}}_u ( \underbrace { {{\boldsymbol{W}}} - {\xi }_1{\nabla }_{\boldsymbol{W}}{\mathcal{L}}_t\left( {{\boldsymbol{W}}},{\boldsymbol{\alpha }} \right)}_{{\boldsymbol{W^{\prime}}}},{\boldsymbol{\alpha }})\\
&&\quad = {\nabla }_{\boldsymbol{\alpha }}{\mathcal{L}}_u\left( {{\boldsymbol{W^{\prime}}},{\boldsymbol{\alpha }}} \right) \\
&&\qquad -\, {\xi }_1{\nabla }_{{\boldsymbol{W^{\prime}}}}{\mathcal{L}}_u\left( {{\boldsymbol{W^{\prime}}},{\boldsymbol{\alpha }}} \right)\nabla _{{\boldsymbol{\alpha }},{\boldsymbol{W}}}^2{\mathcal{L}}_t\left( {{\boldsymbol{W}},{\boldsymbol{\alpha }}} \right)\\
\end{eqnarray*}

In Equation (7), for ease of presentation, we hid the data terms ${D}_v$ and ${D}_t$ in ${\mathcal{L}}_u$ and ${\mathcal{L}}_t$, respectively. To reduce the complexity of Equation (7), the finite difference was used to approximate the second-order derivative:


(8) \begin{eqnarray*}
&&{\nabla }_{\boldsymbol{\alpha }}{\mathcal{L}}_u\left( {{\boldsymbol{W^{\prime}}},{\boldsymbol{\alpha }}} \right) \\
&&\quad -\, {\xi }_1{\nabla }_{{\boldsymbol{W^{\prime}}}}{\mathcal{L}}_u\left( {{\boldsymbol{W^{\prime}}},{\boldsymbol{\alpha }}} \right)\nabla _{{\boldsymbol{\alpha }},{\boldsymbol{W}}}^2{\mathcal{L}}_t\left( {{\boldsymbol{W}},{\boldsymbol{\alpha }}} \right)\\
&& = {\nabla }_{\boldsymbol{\alpha }}{\mathcal{L}}_u\left( {{\boldsymbol{W^{\prime}}},{\boldsymbol{\alpha }}} \right)\\
&&\quad -\, {\xi }_1{\lim }_{\varepsilon \to 0}\frac{{{\nabla }_{\boldsymbol{\alpha }}{\mathcal{L}}_t\left( {{{\boldsymbol{W}}}^ + ,{\boldsymbol{\alpha }}} \right) - {\nabla }_{\boldsymbol{\alpha }}{\mathcal{L}}_t\left( {{{\boldsymbol{W}}}^ - ,{\boldsymbol{\alpha }}} \right)}}{{2\varepsilon }}\\
\end{eqnarray*}

where ${{\boldsymbol{W}}}^ \mp = {\boldsymbol{W}} \mp \varepsilon {\nabla }_{{\boldsymbol{W^{\prime}}}}{\mathcal{L}}_u( {{\boldsymbol{W^{\prime}}},{\boldsymbol{\alpha }}} )$ and $\varepsilon $ denotes the small finite-difference scalar. The second-order derivative in Equation (8) may also be reduced to the first-order derivation ${\nabla }_{\boldsymbol{\alpha }}{\mathcal{L}}_u( {{{\boldsymbol{W}}}^{\boldsymbol{*}}( {\boldsymbol{\alpha }} ),{\boldsymbol{\alpha }}} ) = {\nabla }_{\boldsymbol{\alpha }}{\mathcal{L}}_u( {{\boldsymbol{W}},{\boldsymbol{\alpha }}} )$ when setting ${\xi }_1 = 0$. This first-order approximation is more efficient in computation without sacrificing the accuracy (Fig. S14). Therefore, as otherwise noted, we used the first-order approximation for HIFI learning. Moreover, due to the discontinuity of spike activation function, we adopted the pseudo-derivative approach to solve the issue. In detail, we used the derivative of the triangle function [52]. Please refer to Note S1 for the pseudocode and Fig. S15 for a flow chart.

## Supplementary Material

nwae301_Supplemental_File

## Data Availability

Publicly available data sets were used and referenced with their descriptions in the paper. The neural data used in this paper are available in the following databases: Allen Atlas (https://allensdk.readthedocs.io/en/latest), Paul Manis data set (link: Raw voltage and current traces for current-voltage (IV) relationships for cochlear nucleus neurons) and Shenoy's data set (https://github.com/slinderman/stats320). ScRNA-seq data sets were downloaded from the website page of the ALLEN BRAIN MAP project (https://portal.brain-map.org/atlases-and-data/rnaseq). Source codes in Pytorch are also available at Github (https://github.com/deng-ai-lab/HIFI).
