# Supplementary material for: Biologically inspired heterogeneous learning for accurate, efficient and low-latency neural network
Source: Natl Sci Rev. 2024 Aug 30;12(1):nwae301. doi: 10.1093/nsr/nwae301 (PMC11697980; doi:10.1093/nsr/nwae301)
Supplement: nwae301_Supplemental_File [file nwae301_supplemental_file.pdf]

Supplementary Material

for

Biologically Inspired Heterogeneous Learning for Accurate, Efficient and Low-latency Neural Network

Bo Wang[1,#], Yuxuan Zhang[1,#], Hongjue Li[1], Hongkun Dou[1], Yuchen Guo[3,*], Yue Deng[1,2,*]

[1] *School of Astronautics, Beihang University, Beijing 100191, China.*

[2] *Institute of Artificial Intelligence, Beihang University 100191, Beijing, China.*

[3] *Institute for Brain and Cognitive Sciences, BNRist, Tsinghua University 100084, Beijing, China.*

[#] These authors contributed equally to this work.

[*] Correspondence: yuchen.w.guo@gmail.com (Y.G.), ydeng@buaa.edu.cn (Y.D.)

Supplementary Note 1. HIFI model ... 1
HIFI objective ... 1
HIFI architecture ... 1
HIFI optimization process ... 2
Supplementary Note 2. Explanation of the bi-level programming paradigm ... 3
Structure Description ... 3
Mathematical Definition ... 3
Rationale for Use in the HIFI Model ... 3
Illustrative Example with HIFI ... 3
Supplementary Note 3. Reproducing neural statistics of real biological neurons in mouse brain ... 5
Neural recordings ... 5
Reproduce Neural statistics of biological neurons ... 5
Evaluation metric ... 6
Supplementary Note 4. Classification on image datasets ... 7
Handwritten digit recognition ... 7
CIFAR10 / CIFAR100 recognition ... 7
ImageNet recognition ... 7
Evaluation metric ... 7
Supplementary Note 5. Classification on neuromorphic datasets ... 9
N-MNIST Digit classification ... 9
DVS128-Gesture classification ... 9
DVS-CIFAR10 classification ... 9
SHD and SSC audio classification ... 9
Supplementary Note 6. Cell Type Identifications from scRNA-seq ... 10
Datasets ... 10
Data preprocessing ... 10
Model evaluation across different levels of annotations ... 10
Model evaluation across species ... 10
Architecture and training settings ... 10
Supplementary Figures ... 11
Supplementary Tables ... 26
Supplementary References ... 36

Supplementary Note 1. HIFI model

HIFI objective

HIFI contains two sets of learnable parameters, i.e., the network-level synapse weight set $\boldsymbol{W} = \{\boldsymbol{W}^k\}$ and the neuron-level biophysical parameters set $\boldsymbol{\alpha} = \{\boldsymbol{\alpha}^k\}$. These two sets of parameters work together for controlling the information encoding and learning processes of the whole neural network. Here, by observing the hierarchy of learnable variables $\boldsymbol{W}$ and $\boldsymbol{\alpha}$**,** we introduced a bi-level programming[1, 2] paradigm for HIFI learning,

$$\min_{\boldsymbol{\alpha}} \mathcal{L}_v(D_v; \boldsymbol{W}^*(\boldsymbol{\alpha}), \boldsymbol{\alpha}) + \lambda\Omega(\boldsymbol{W}^*(\boldsymbol{\alpha}), \boldsymbol{\alpha})$$
$$s.t.\ \boldsymbol{W}^*(\boldsymbol{\alpha}) = \operatorname*{argmin}_{\boldsymbol{W}} \mathcal{L}_t(D_t; \boldsymbol{W}, \boldsymbol{\alpha}). \tag{S1}$$

The above learning problem is a bi-level optimization because $\boldsymbol{W}$ is a constraint function of $\boldsymbol{\alpha}$ by solving the minimization of the network-level objective in the constraint. In detail, the network-level objective for synapse connection ($\boldsymbol{W}$) learning is the empirical loss evaluating the performance of HIFI on training data D_t with the neuron-wise parameters ($\boldsymbol{\alpha}$) returned by the neuron-level loop. The first term $\mathcal{L}_v(\cdot)$ in the neuron-level objective defines the empirical loss of HIFI on validation data D_v with the currently learned parameters $\boldsymbol{W}^*(\boldsymbol{\alpha})$ and $\boldsymbol{\alpha}$. The second term, $\Omega(\cdot)$ is the regularization term that incorporates the Laplacian smoothness for encouraging neighboring neurons to share similar neuron-level parameters. To be specific, self-inhibiting neurons with neuron-level parameters (node feature) and synapses with different weights (edge weight) are regarded as nodes and edges of a weighted graph $\mathbb{G}$, respectively. The features of all nodes span a feature matrix $\boldsymbol{F} = [\boldsymbol{\alpha}^1, \boldsymbol{\alpha}^2, \ldots, \boldsymbol{\alpha}^K]^T \in \mathbb{R}^{K\times 5}$ with each column $\boldsymbol{\alpha}^k \in \mathbb{R}^5$ representing neuron-level parameters of the k-th neuron node. Then, the regularization term was formulated as

$$\Omega(\boldsymbol{W}, \boldsymbol{\alpha}) = tr(\boldsymbol{F}^{\boldsymbol{T}}\boldsymbol{L}\boldsymbol{F}). \tag{S2}$$

In the above equation, $\boldsymbol{L} = \boldsymbol{D} - \boldsymbol{A}$ is the Graph Laplacian matrix, where $\boldsymbol{D}$ is a degree matrix by summing up each row of $\boldsymbol{A}$ at diagonal, and $\boldsymbol{A}$ is the weighted adjacency matrix where $\boldsymbol{A}_{i,j} = \boldsymbol{W}(i,j)$ denotes the weight for edge (i,j) if $i{\sim}j$ and $\boldsymbol{A}_{i,j} = 0$ otherwise. To facilitate the computation, the Laplacian smoothness regularization was imposed on each single feature map independently.

HIFI architecture

The HIFI can share the same architecture as ANNs with differences on the usage of different types of neuron models and network heterogeneity (**Fig. S1**). The neuron model used in ANNs can be represented as $y = f(\boldsymbol{W}\boldsymbol{X} + b)$, where y is the neuron output, f represents the activation function, $\boldsymbol{W}$ is the weight vector of the neuron, $\boldsymbol{X}$ is the input vector of the neuron, and b is the bias. For HIFI, the neuron model is replaced by our designed self-inhibiting neuron model as described in equation (2). Meanwhile, all neurons in ANNs share the prefixed and homogenous intra-neuron systems (e.g. ReLU), however, neurons in HIFI can own their unique intra-neuron systems after heterogeneous learning. For example, a fully-connected architecture 784-250-10 can

be configured by both ANNs and HIFI with the differences on neuron models and heterogeneity (**Fig. 3a**).

HIFI optimization process

To elucidate the optimization process of HIFI, we present the pseudocode and an optimization process flowchart (**Fig. S15**).

Pseudocode of HIFI Optimization

Input: Training data D_t, Validation data D_v, Learning rate ξ_1 at the network level, Learning rate ξ_2 at the neuron level

Output: $\boldsymbol{W}_{best}$, $\boldsymbol{\alpha}_{best}$

Initialize $\boldsymbol{W}$ and $\boldsymbol{\alpha}$;

While not converged **do**

Step 1: $\boldsymbol{\alpha} \coloneqq \boldsymbol{\alpha} - \xi_2 \cdot \nabla_{\alpha}\mathcal{L}_v(D_v; \boldsymbol{W} - \xi_1 \nabla_w \mathcal{L}_t(D_t; \boldsymbol{W}, \boldsymbol{\alpha}), \boldsymbol{\alpha})$ with Adam gradient descent (first approximation when $\xi_1 = 0$, second approximation when $\xi_1 \neq 0$) /* $\boldsymbol{\alpha}$ optimization

Step 2: $\boldsymbol{W} \coloneqq \boldsymbol{W} - \xi_1 \nabla_W \mathcal{L}_t(D_t; \boldsymbol{W}, \boldsymbol{\alpha})$ with SGD gradient descent /* $\boldsymbol{W}$ optimization

end

Supplementary Note 2. Explanation of the bi-level programming paradigm

Structure Description

The bi-level programming paradigm is an optimization framework that consists of two nested levels of optimization problems[3]. The upper-level problem (leader) sets the objectives and constraints, while the lower-level problem (follower) performs optimization within these constraints. The specific structure is as follows:

Upper-Level Problem (Leader).

$$\begin{array}{c}\text{Objective function: } F(x, y) \\ \text{Constraints: } G(x, y) \leq 0.\end{array} \tag{S3}$$

Lower-Level Problem (Follower).

$$\begin{array}{c}\text{Objective function: } f(x, y) \\ \text{Constraints: } g(x, y) \leq 0.\end{array} \tag{S4}$$

The solution x of the upper-level problem influences the optimization process of the lower-level problem, and the solution y of the lower-level problem, in turn, affects the objective function of the upper-level problem.

Here, $F(x, y)$ and $f(x, y)$ represent the objective functions for the upper and lower-level problems, respectively, while $G(x, y) \leq 0$ and $g(x, y) \leq 0$ are their constraints. The solution x of the upper-level problem influences the optimization process of the lower-level problem, and the solution y of the lower-level problem, in turn, affects the objective function of the upper-level problem.

Mathematical Definition

The bi-level programming problem can be formalized as:

$$\begin{array}{c}\min_{x} F(x, y) \\ s.t. \mathrm{y} = \operatorname{argmin} \mathrm{f(x, y)}.\end{array} \tag{S5}$$

Here we let X represent the feasible region for the upper-level decision variable x, and Y(x) represent the feasible region for the lower-level decision variable y dependent on x. To ensure the existence of a solution, the feasible regions X and Y(x) must be non-empty. The uniqueness of the lower-level solution y for a given x is crucial for the well-defined nature of the upper-level problem. This often necessitates the convexity of the lower-level problem.

Rationale for Use in the HIFI Model

In the HIFI model, the bi-level programming paradigm is employed to achieve holistic optimization by separately handling high-level network parameters and detailed parameter settings. Specifically, the upper-level optimization focuses on the neuron-level biophysical parameters $\boldsymbol{\alpha}$, while the lower-level optimization deals with the network-level synapse weights $\boldsymbol{W}$. This hierarchical optimization ensures that the overall network structure is optimized first, followed by fine-tuning specific parameters, leading to more efficient and effective performance.

Illustrative Example with HIFI

Consider a scenario within the HIFI model where the objective is to optimize the overall network performance. The upper-level problem focuses on optimizing the neuron-level biophysical parameters $\boldsymbol{\alpha}$ to achieve the best empirical loss on validation data D_v. Once $\boldsymbol{\alpha}$ is optimized, the lower-level problem addresses the optimization of the network-level synapse weights $\boldsymbol{W}$ to minimize the empirical loss on training data D_t. Based on the aforementioned description of bi-level optimization, the HIFI model can be formally represented as Eq. S1. This structured approach allows for an efficient and flexible optimization process, ensuring the HIFI model's overall network performance is maximized.

Supplementary Note 3. Reproducing neural statistics of real biological neurons in mouse brain

Neural recordings

Electrophysiological recordings data. The electrophysiology data of Htr3a neuron (specimen ID 474637203) in Allen Cell Type Database[4] (link is shown in the *Data and code availability*) was used to evaluate the performance of neuron models. Long square stimuli (sweep 56 and 57) and noise1 stimuli (sweep 62, 66 and 68) were separately utilized to optimize and test neuron models.

Electrophysiology and behavioral data. The dataset from Shenoy's lab at Stanford University consists of recordings from a 96-channel Utah array implanted in the motor cortex of a non-human primate engaged in a center-out-and-back reaching task[5]. The data is not spike sorted and simply records the timing of presumptive spikes detected on each channel of the Utah array. It contains a total of 500 trials, of which 400 are reserved for training and validation and 100 for testing. Each trial features a (T, 96) array representing spike counts—where T denotes the number of time bins—a (T, 2) array of cursor velocities, and a label indicating the specific reaching condition (8 center-out and 1 out-center). To improve the signal-to-noise ratio, the spike and position data initially recorded in 1ms bins are down sampled to 25ms bins.

Reproduce Neural statistics of biological neurons

Electrophysiological recordings data. The artificial neuron model received and integrated the injected currents from the electrophysiology data, and outputted the spike trains. Surrogate gradient method was used to train the neuron models. The training settings were shown in **Table S5**. The long-square recordings of sweep 57 and 56 were separately used as the training and validation dataset, and the noise recordings of sweep 62, 66 and 68 were used to evaluate the ability of neuron models to reproduce neuronal spike times.

Ablation study. The LIF neuron model in the electrophysiological experiment holds the same neural dynamics except for the self-inhibiting autapse. And we compared the performance of reproducing the neuronal spike times between LIF and self-inhibiting neuron model to investigate the importance of self-inhibiting autapse in our model.

Augmented data generation.

The process of data augmentation involves three main stages: template selection, spiking generator training, and data generation. For template selection, the dataset consists of 500 trials, each corresponding to one of nine reaching conditions (8 center-out and 1 out-center) with spike patterns of the same condition exhibiting a consistent distribution. To minimize the training complexity and instability, a single biological spike pattern is selected as the template ground truth for each reaching condition, following the SNN_EEG[6] approach. For spiking generator training, SNNs were used to generate neuronal activation patterns in response to external stimuli, with a four-layer (96-512-1024-512-96) fully-connected structure. LIF, iLIF, SNU and self-inhibiting neuron model were used within the SNNs, respectively. A fixed Poisson spike train served as the input stimuli for each reaching condition to replicate the selected template, with training guided by the mean square error

(MSE) loss function. For data generation, the trained generator used fixed Poisson spike trains, supplemented with additional Gaussian perturbations, to produce diverse and biologically plausible spike trains for each reaching condition. These generated spike trains share the same cursor velocities as the corresponding template patterns. The training settings were shown in **Table S6**.

Evaluation of augmented data with BCI decoders. Brain-computer interface (BCI) decoders were utilized to predict the corresponding velocities at different time steps based on biological spike trains input. To assess the quality of the augmented data, we compared BCI decoders trained with and without generated data to measure the biological fidelity of the augmentation. Specifically, GRU, LSTM and 1D CNN were separately used as decoders, with MSE loss guiding the learning of velocities. To visualize the decoder's predictions, the cursor velocities were cumulatively integrated and Gaussian smoothed to derive the cursor trajectory. The R^2 score was then calculated to estimate the similarity between the decoded trajectory and the ground truth.

Evaluation metric

Macro F1-score. The macro F1-score, giving equal weight to every category regardless of its frequency, is often used to evaluate the imbalanced data. The macro F1-score is formulated as $\text{Macro F1} = \frac{1}{n}\sum_{i=1}^{n} \text{F1}_i$, where n is the number of classes, and F1_i denotes the F1-score of class i. The F1_i is defined as $\text{F1}_\text{i} = 2R_iP_i/(R_i + P_i)$, where R_i denotes the recall of class i and the P_i denotes the precision of class i.

Normalized, bias-corrected metrics M_d^*. The similarity M_d^* between two sets of spike trains is defined as[7, 8],

$$M_d^* = \frac{2 \cdot \langle v_d, v_m \rangle}{\frac{2}{N_d(N_d - 1)} \sum_{i=1}^{N_d} \sum_{i'=i+1}^{N_d} \langle S_i^{(d)}, S_{i'}^{(d)} \rangle + \langle v_m, v_m \rangle} \tag{S6}$$

where $S_i^{(d)}$ is the experimental spike trains recorded in response to N_d repetitive injections, $\text{i} = 1, \ldots, N_d$.of the same input current $I_{test}(t)$, and $S_i^{(m)}$ is the reproduced spike trains of neuron models by N_m repetitions.$v_d = \frac{1}{N_d}\sum_{i=1}^{N_d} S_i^{(d)}$ is the average experimental recordings across trials, $v_m = \frac{1}{N_m}\sum_{i=1}^{N_m} S_i^{(m)}$ is the average reproduced spikes and $\langle v_m, v_m \rangle$ is the norm. $\langle S_i^{(d)}, S_{i'}^{(d)} \rangle = \int_0^T \int_{-\infty}^{\infty} \int_{-\infty}^{\infty} K(s, s') S_i(t - s) S_j(t - s') ds ds' dt$ represents the inner product between two spike trains, and $K(s, s')$ is the Kistler coincidence kernel defining the degree of coincidence between two spikes occurred at times s and s'.

Supplementary Note 4. Classification on image datasets

Handwritten digit recognition

The MNIST[9] dataset consists of single-channel grayscale images from 10 classes ('0' to '9') in the resolution of 28×28 pixels. The whole dataset is split into 60,000 training data and 10,000 testing data. A fully connected architectures with 784-250-10 neurons was used for MNIST classification. Detailed training settings were illustrated in **Table S7**.

CIFAR10 / CIFAR100 recognition

The CIFAR10 dataset consists of 60,000 32×32 color images in 10 classes. The whole dataset is divided into 50,000 training and 10,000 testing samples. Cutmix and auto-augmentation were used following the official pytorch image classification reference training scripts. Our implement used the SLTT[10] framework. For energy evaluation on CIFAR10, four classical architectures were utilized, including AlexNet, CifarNet, VGG-9 and ResNet-18. The detailed architecture and training settings were depicted in **Table S7**. The CIFAR100 dataset has 100 classes containing 600 images each. The data augmentation strategy was the same as CIFAR10. ResNet-18 was adopted for the CIFAR100 classification, and the detailed training settings were represented in **Table S7**.

ImageNet recognition

The ImageNet dataset (ILSVRC 2012 version) features over 1.2 million high-resolution training images categorized into 1,000 classes. We used the SEW-ResNet-34[11] for classifying the ImageNet dataset, and the detailed training settings were represented in **Table S7**.

Evaluation metric

Accuracy, inference speed, average spikes per neuron, energy consumption on CMOS, and normalized energy consumption in neuromorphic architectures were used to evaluate models.

Accuracy. The accuracy is formulated as $accuracy = \frac{\sum_i \mathbf{1}(y_i = \hat{y}_i)}{n}$, where y_i is the prediction of sample i, $\hat{y}_i$ is the ground truth of sample i, and n is the number of samples.

Inference speed. The speed is formulated as $IS = 1/t$, where the t is represented as the number of time steps.

Energy efficiency (on CMOS). The energy consumption is calculated by the total number of floating-point operations[12, 13] (FLOPS). For ANN, the FLOPS of the l-th layer are calculated by[12]

$$\begin{aligned} F_l^{ANN} &= {k_l}^2 \times H_l \times W_l \times C_{l,i} \times C_{l,o}, \quad \text{Convolutional layer} \\ F_l^{ANN} &= f_{l,i} \times f_{l,o}, \quad \text{Fully-connected layer} \end{aligned} \tag{S7}$$

where k_l is the kernel size, H_l and W_l are the height and width of the feature map, $C_{l,i}$ and $C_{l,o}$ are the input and output channels of the l-th convolutional layer, and $f_{l,i}$ and $f_{l,o}$ are input and output features of the l-th fully-connected layer. Similarly, the FLOPS of the l-th layer of SNNs

are calculated by[13],

$$
\begin{aligned}
F_l^{SNN} &= k_l^2 \times H_l \times W_l \times C_{l,i} \times C_{l,o} \times \lambda_l, \ \text{Convolutional layer} \\
F_l^{SNN} &= f_{l,i} \times f_{l,o} \times \lambda_l, \qquad \text{Fully-connected layer}
\end{aligned}
\tag{S8}
$$

where the average spiking activity λ_l is the ratio between the number of total spikes and the number of neurons of the l-th layer.

The neural networks mainly perform the floating-point (FP) multiply-accumulate (MAC) operations and FP addition operations. On a standard 45nm CMOS process, the energy consumption is 4.6 pJ for a 32bit FP-MAC operation and 0.9 pJ for the FP-addition operation[14-16]. The energy consumption of SNNs is also often evaluated in fixed-point operation[13]. The energy consumption for the fixed-point representations is 3.2 pJ for MAC operation and 0.1 pJ for addition operation. In ANN, all layers operate the FP-MAC operations. In SNN, only the first layer performs the MAC operations while the remaining layers operate the addition operations. Accordingly, energy consumption of SNNs and ANNs can be quantified by,

$$
\begin{aligned}
E_{ANN} &= \sum_{l=1}^{L} F_l^{ANN} \times 4.6 \\
E_{SNN} &= F_l^{SNN} \times 4.6(3.2) + \sum_{l=2}^{L} F_l^{SNN} \times 0.9(0.1)
\end{aligned}
\tag{S9}
$$

Normalized energy consumption on neuromorphic architecture. For the energy consumption of SNN implemented on the neuromorphic architecture, both dynamic (E_{dyn}) and static (E_{sta}) energy should be considered. To be specific, we calculate the energy with latency (static energy E_{sta}) and spikes (dynamic energy E_{dyn}). The normalized energy can be estimated as

$$
E_N = \#Spikes \times E_{dyn} + \#Timesteps \times E_{sta} \tag{11}
$$

where E_{dyn} and E_{sta} are defined as 0.4 and 0.6 on TrueNorth, 0.64 and 0.36 on SpiNNaker.

Supplementary Note 5. Classification on neuromorphic datasets

We used two video neuromorphic datasets, N-MNIST and DVS128-Gesture, and two audio neuromorphic datasets, SHD (Spiking Heidelberg Digits) and SSC (Spiking Speech Commands). The detailed training settings were listed in **Table S8**.

N-MNIST Digit classification

The N-MNIST[17] is a spiking dataset using a Dynamic Vision Sensor (DVS) moving on a pan-tilt unit of the MNIST dataset, consisting of 60,000 training samples and 10,000 testing samples from 10 classes ('0' to '9'). Each sample contains 'on' and 'off' spikes and is resolved by 34×34 pixels ranging within 300ms. All the SNNs were configured in a 12C5-P2-64C5-P2 architecture.

DVS128-Gesture classification

The DVS128-Gesture dataset[18] contains 1,342 instances of 11 different actions collected from 29 different individuals using the DVS128 camera under 3 different lightening conditions. The architecture of SNNs for this task was defined as P4-64C3-128C3-P2-128C3-P2-256.

DVS-CIFAR10 classification

DVS-CIFAR10[19] contains 10,000 frame-based images coming from CIFAR-10, whose resolution is 128×128 pixels. The dataset has an intermediate difficulty with 10 different classes. We used the CifarNet architecture to evaluate the performance of HIFI in DVS-CIFAR10.

SHD and SSC audio classification

The Spiking Heidelberg Digits[20] (SHD) contains 10,420 recordings of spoken digits ranging from 0 to 9 in English and German language, which are collected from 12 individuals. The Spiking Speech Command[20] (SSC) dataset consists of 105,829 audio recordings of 24 single word commands (Yes, No, One, Two, Three, etc.) in the English language, which are collected from 1864 speakers. Both datasets comprise audio data converted into spike trains based on a detailed ear model. We used the same neural network architecture[20] in the paper publishing these datasets.

Supplementary Note 6. Cell Type Identifications from scRNA-seq

Datasets

Four scRNA-seq datasets were used in this study including Allen Mouse Brain (AMB), Mouse V1, Mouse ALM (GSE115746), and Human MTG (phs001790).

Data preprocessing

The cell ranger of scanpy[21] were used to filter out Top 5000 genes with highest variability. Then, gene expressions of neurons were processed by CPM read count normalization. Finally, the counts were increased by 1, normalized by log_e and scaled by min-max normalization.

Model evaluation across different levels of annotations

In the whole AMB dataset, we filtered out cell types covering less than 10 cells and got two levels of annotations, e.g. AMB16 and AMB92, respectively. The training and testing data were splitted by 5-fold cross validations. The classification accuracy and macro F1-score were used as the evaluation metric.

Model evaluation across species

The Mouse ALM, Mouse V1, Human MTG, were utilized to compose three cross-species datasets including ALM-MTG, V1-MTG, ALM+V1-MTG. The datasets before and after the dash were respectively used as the training and testing datasets. Only overlapped genes in both datasets were used.

Architecture and training settings

We used the same neural network architectures suggested in the ACTINN and configured all SNNs in the structure of input-100-50-25-output. The detailed settings for HIFI training were shown in **Table S9**. The STBP and SNU were trained by SGD optimizer with initial learning rate 0.01, batch size 128, time step 40 and weight decay 5×10^{-4}.

Supplementary Figures

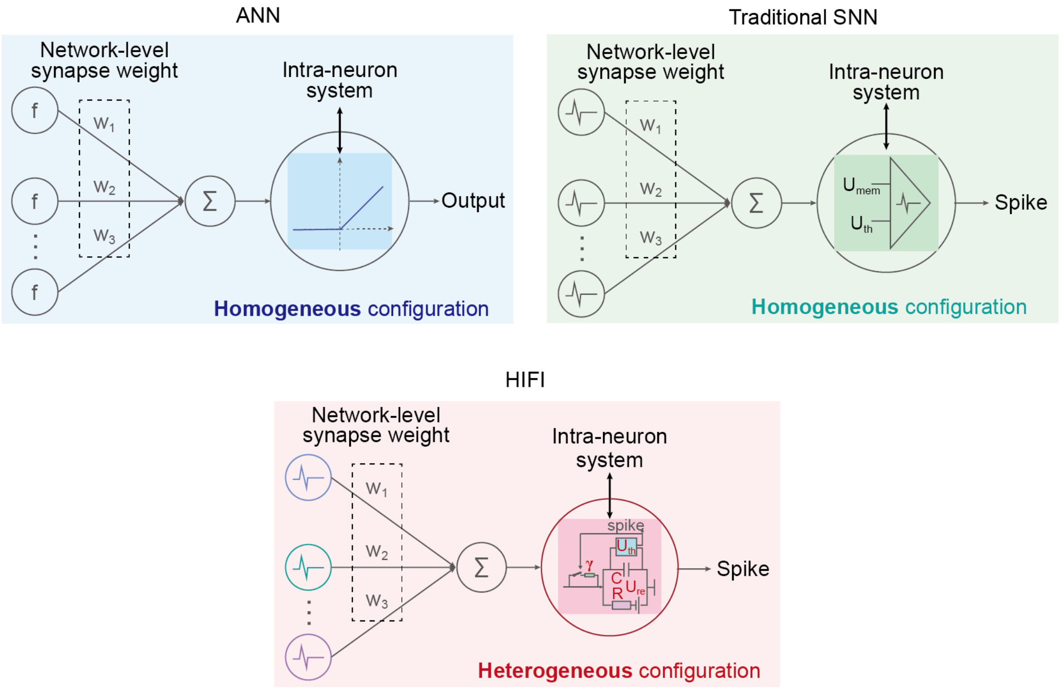

Supplementary Figure 1 | The conceptual comparisons of ANN, tradition SNN and HIFI.

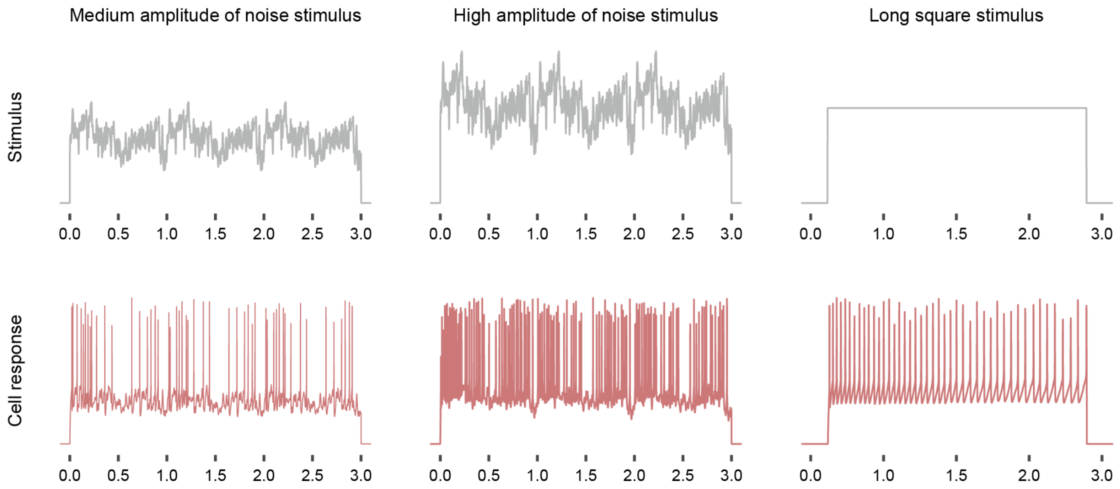

Supplementary Figure 2 | Three types of incurrent injections and corresponding responses of the biological neuron, Htr3a.

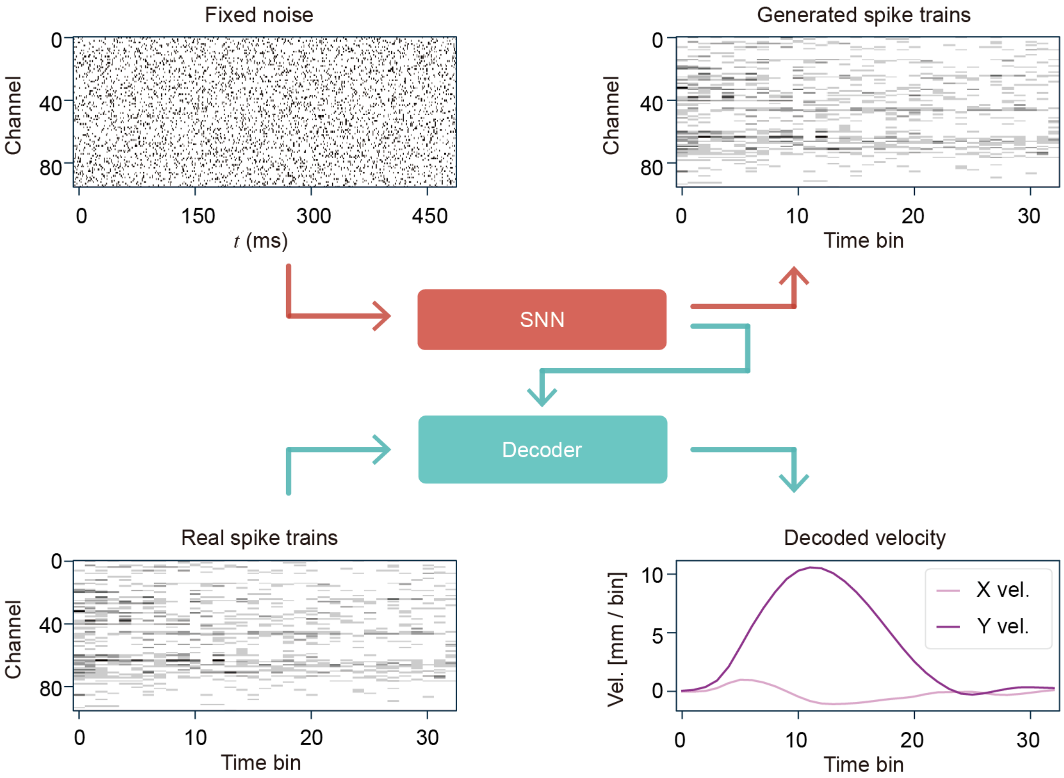

Supplementary Figure 3 | Pipeline of neural recording simulation using SNNs. The diagram shows the training (top) and evaluation (bottom) phases. Low-cost noise inputs to SNNs produce simulated neural recordings that are later used to augment the training process of downstream BCI decoder.

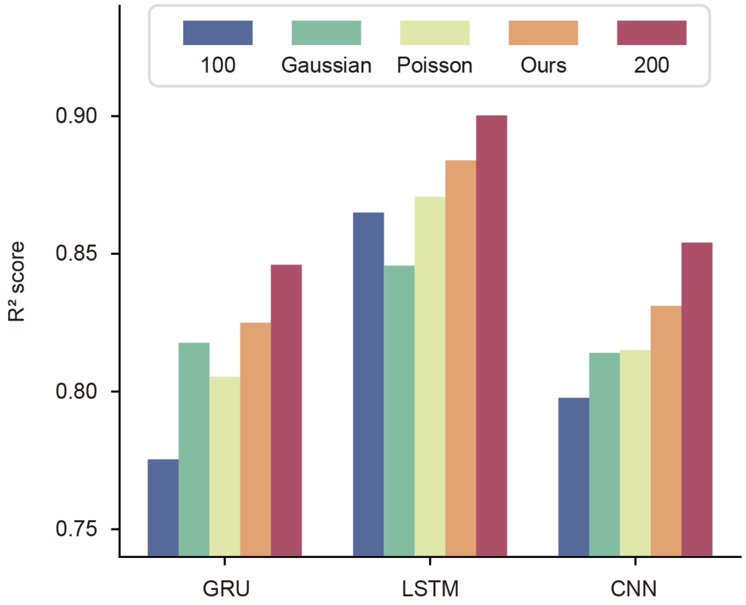

Supplementary Figure 4 | R^2 scores with or without data augmentation are compared on various trained decoders. 100: Real neural data samples (N=100); **200:** Real neural data samples (N=200); **Gaussian:** 100 real samples + 100 augmented samples by adding Gaussian noise to real neural data samples; **Poisson:** 100 real samples + 100 augmented samples by adding Poisson noise to real neural data samples; **Ours:** 100 real samples + 100 augmented samples generated by SNNs with our self-inhibiting neuron

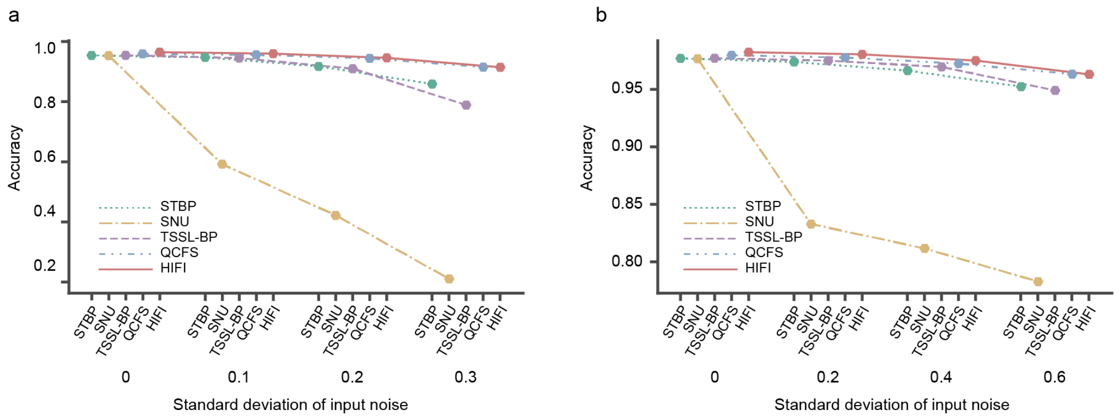

Supplementary Figure 5 | Robustness evaluation on MNIST by the perturbations of Gaussian noise (left) and speckle noise (right). Additive Gaussian noise is sampled from a Gaussian distribution, $\mathcal{N}_1(0, \sigma^2)$. Additive speckle noise is modeled by random value multiplications with pixel values of images, where the random values are sampled from a uniform distribution, $\mathcal{N}_2(0, \sigma^2)$.

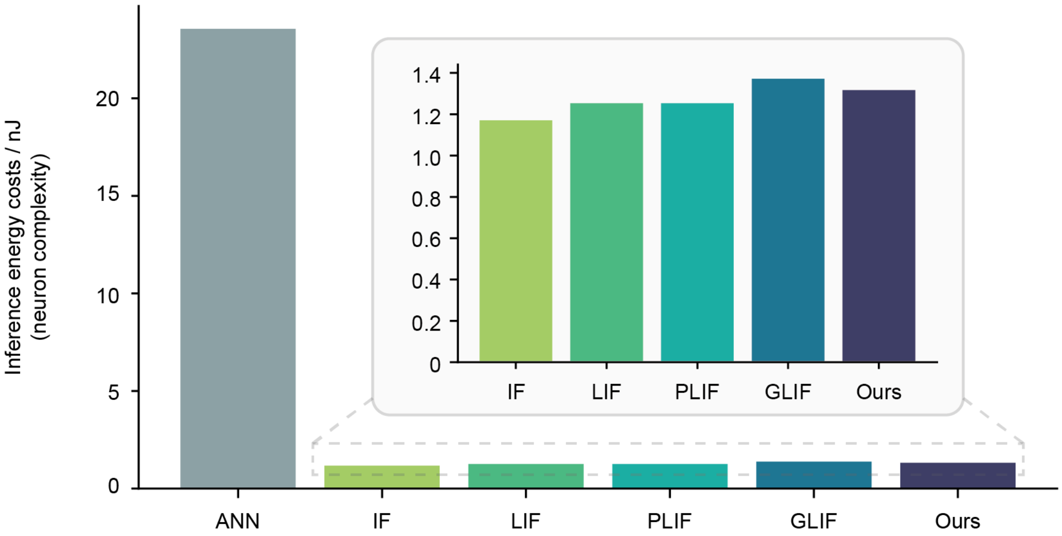

Supplementary Figure 6 | Comparison of operational complexity across different neuron models. The operational complexity (evaluated by the inference energy costs) of ANN and SNNs composed of different neuron models, including IF, LIF, PLIF, GLIF, and our proposed self-inhibiting neuron model on a 512-10 dense layer ($m = 512,\ n = 10,\ \mathrm{Fr}_{\mathrm{in}} = 0.25,\ E_{\mathrm{AC}} = 0.9\ pJ$, $E_{\mathrm{MAC}} = 4.6\ pJ$).

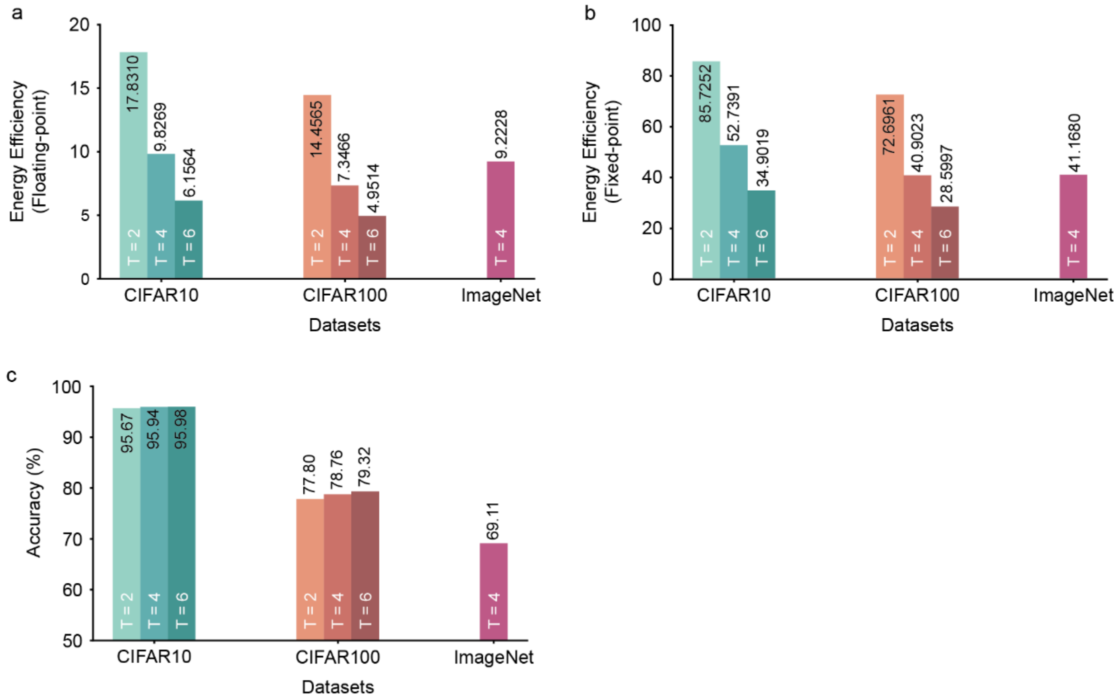

Supplementary Figure 7 | Energy efficiency improvements between HIFI and ANN across different datasets and latencies. (**a**), Energy efficiency (floating-point representation) and **(b)**, Energy efficiency (fixed-point representation) are shown for CIFAR10, CIFAR100, and ImageNet datasets with latencies T = 2, T = 4, and T = 6. The bars illustrate that HIFI significantly reduces energy consumption compared to ANN, especially at lower latencies.

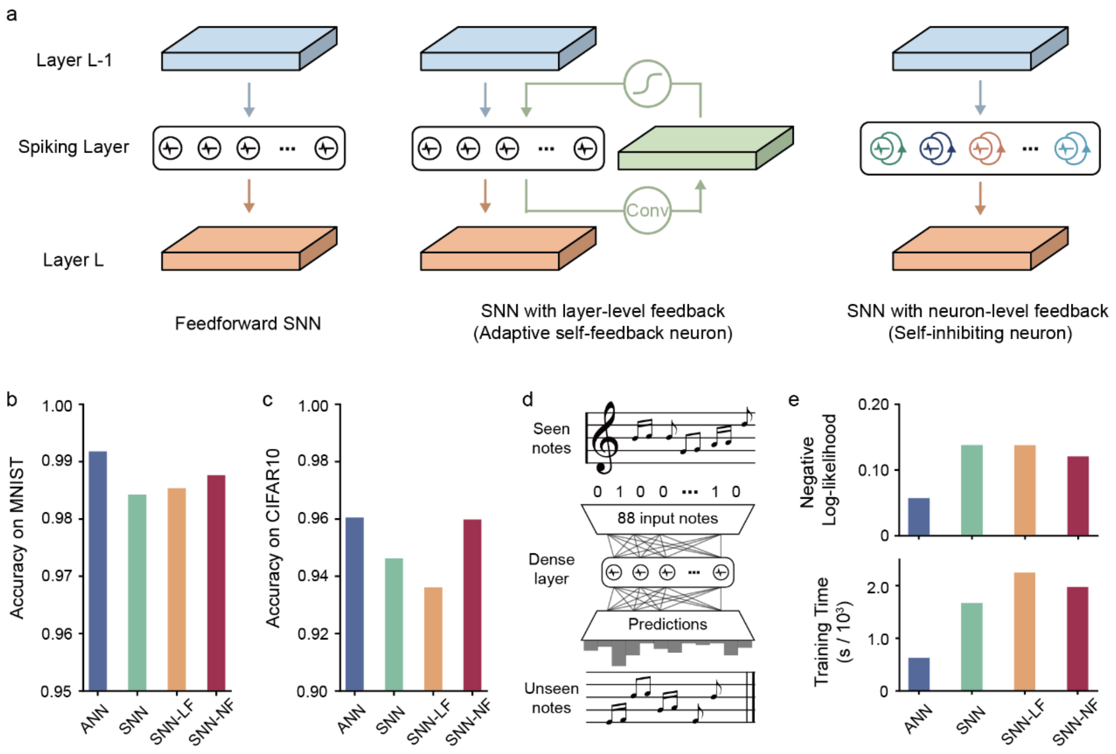

Supplementary Figure 8 | Comparisons of SNNs with the self-inhibiting neuron and the learnable inter-layer feedback connections. (**a**), Schematic diagrams of a common SNN (left), an SNN with learnable inter-layer feedback (middle), and an SNN with the self-inhibiting neuron (right). (**b-c**), Accuracy comparisons of ANN and SNNs with different types of feedback connections on MNIST and CIFAR10. **SNN**: Feedforward SNNs without feedback connections. **SNN-LF**: SNNs with inter-layer feedback connections. **SNN-NF**: SNNs with neuron-level feedback connections, i.e. the self-inhibiting neurons. (**d**), Music prediction: the model predicts the probabilities of the consecutive notes to be played. (**e**), The performance and training cost comparisons of various models in music prediction.

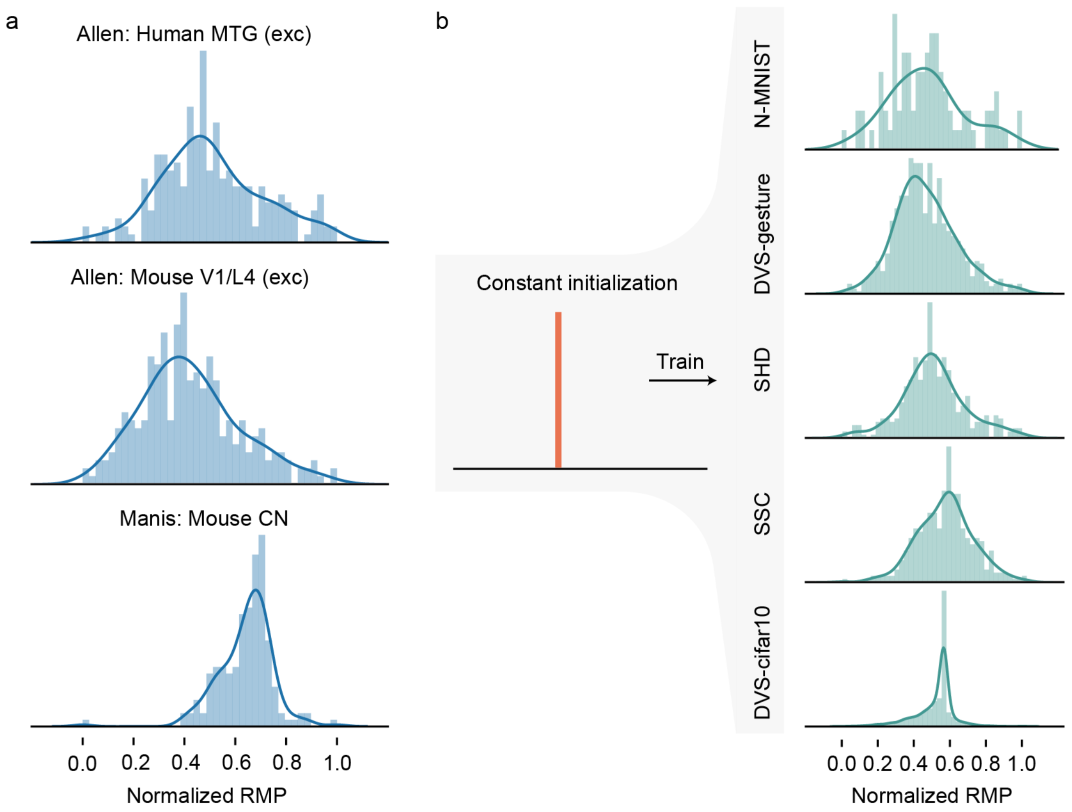

Supplementary Figure 9 | (**a**) Experimentally observed distributions of resting membrane potential (RMP) for human middle temporal gyrus, mouse V1 layer 4, and mouse cochlear nucleus. (**b**) Resting membrane potential distributions before (left) and after (right) training for each neuromorphic dataset.

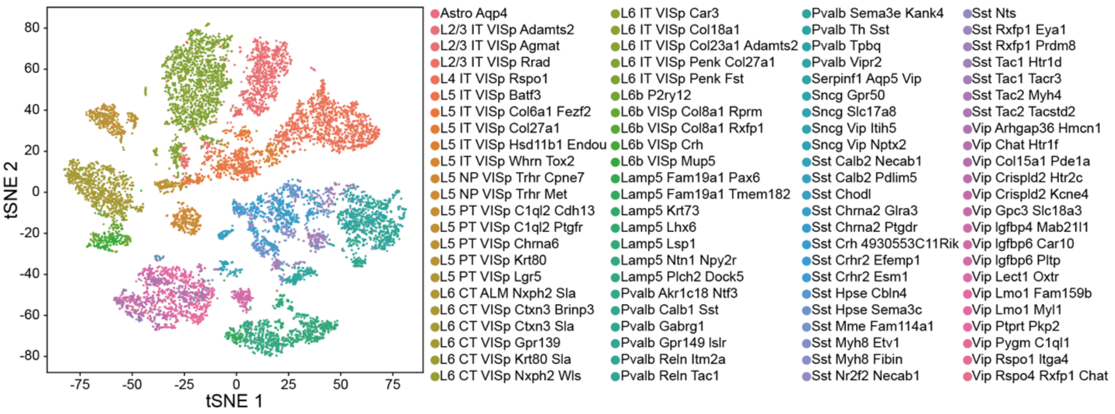

Supplementary Figure 10 | tSNE visualizations of AMB92.

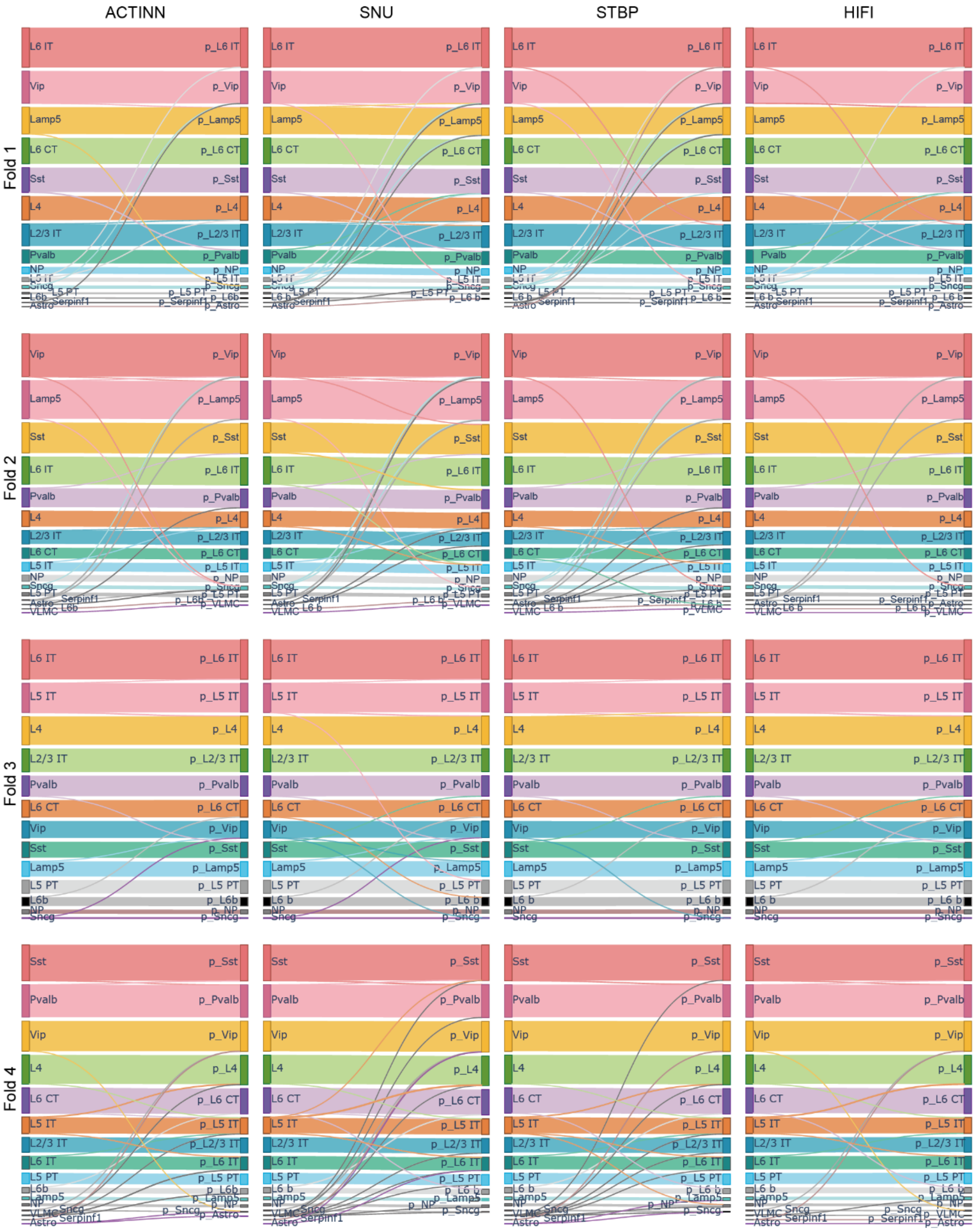

Supplementary Figure 11 | Sankey plots for data in other folds of AMB16. Sankey plot: cell numbers of each ground truth cell type (left nodes), cell flows from ground truth to predictions (middle link), cell numbers of each predicted cell type (right nodes).

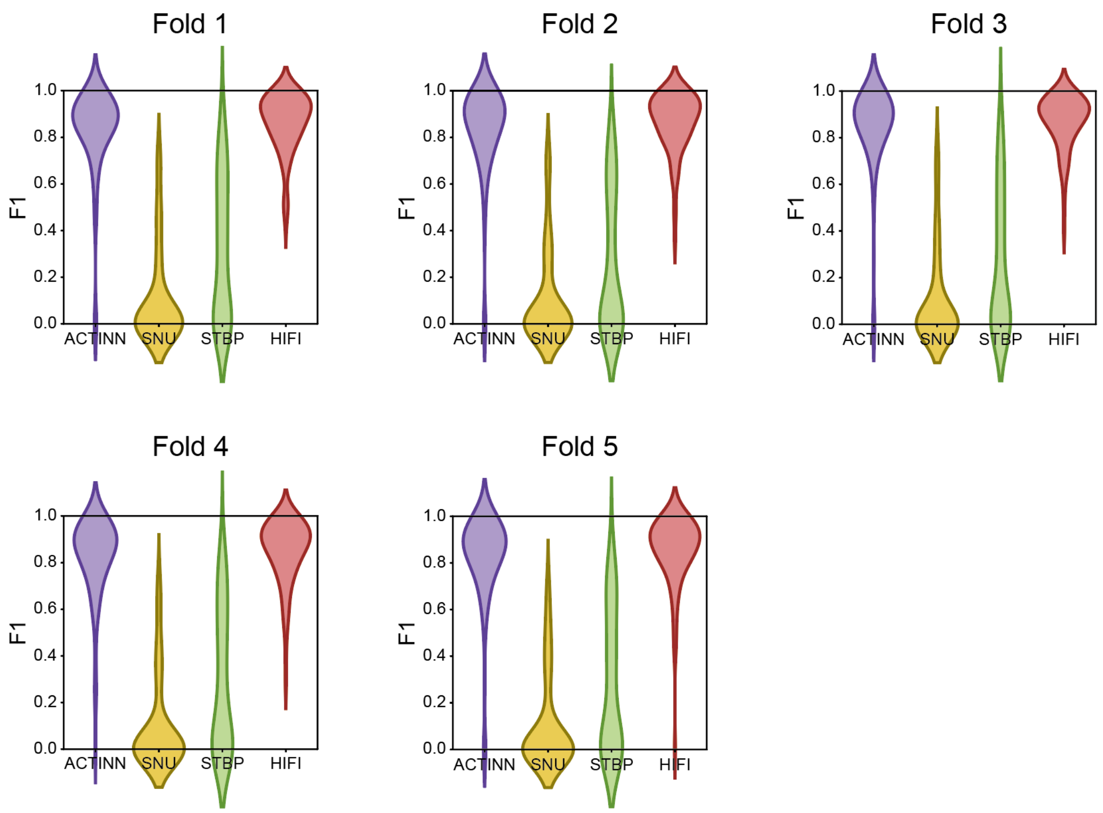

Supplementary Figure 12 | Violin plot of F1-scores of all data of AMB92. Each violin plot shows the distribution of F1 scores of all the 92 cell types in AMB92.

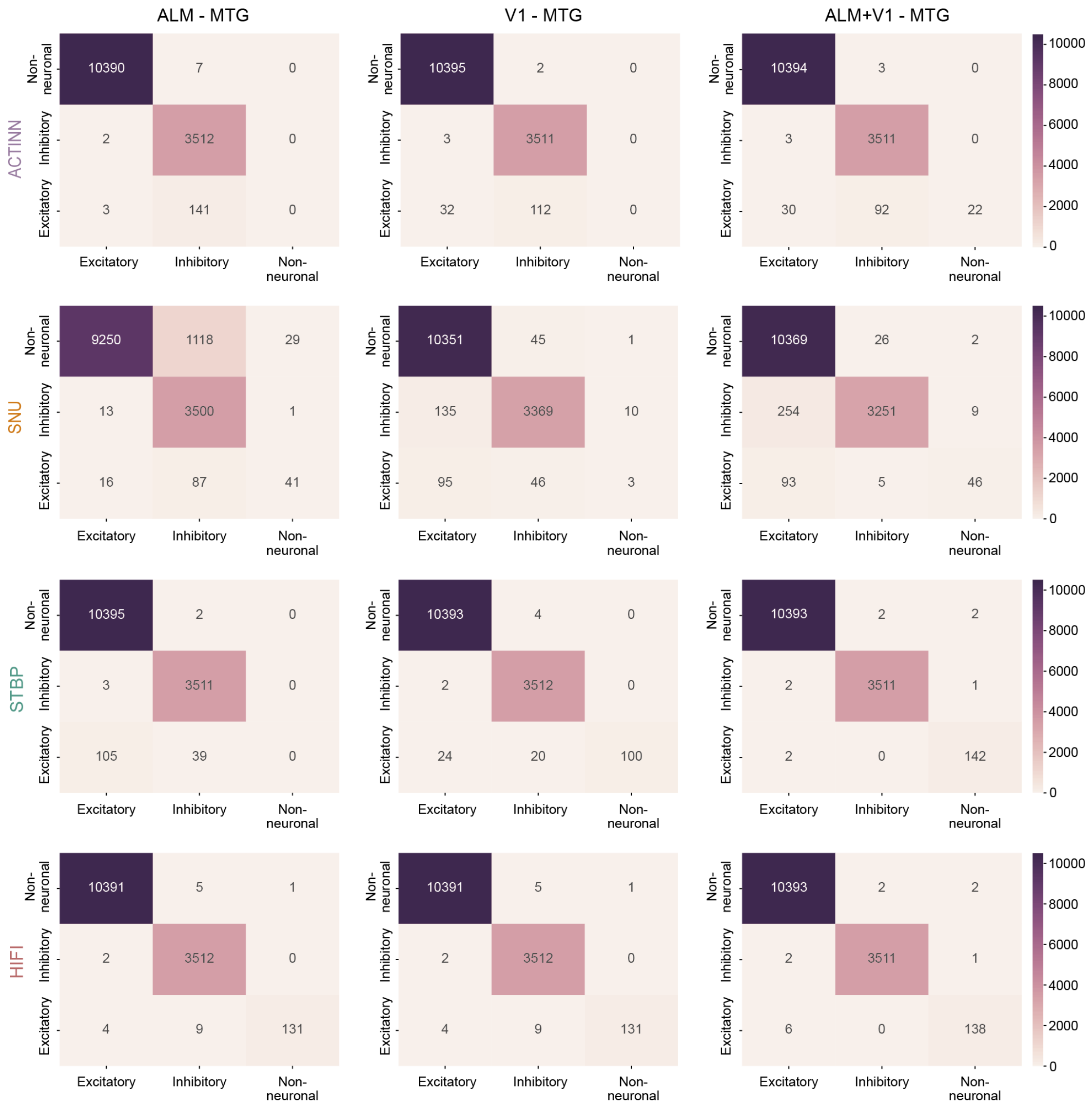

Supplementary Figure 13 | Performance of different models in the across-species tests. Each confusion matrix shows the detailed predictions of different models (ACTINN, SNU, STBP, and HIFI) for different dataset pairs (ALM-MTG, V1-MTG, and ALM+V1-MTG).

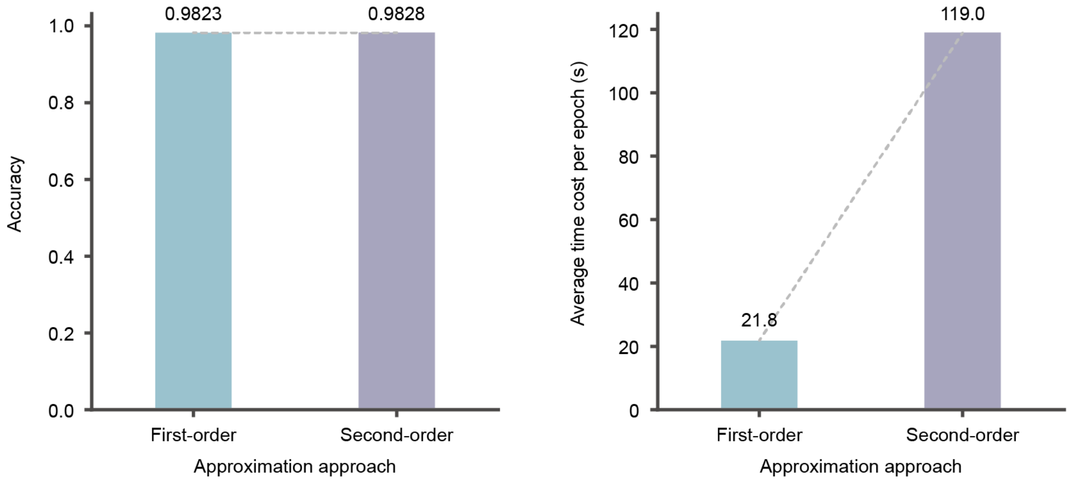

Supplementary Figure 14 | The performance and run time of HIFI on MNIST dataset with first-order and second-order approximation.

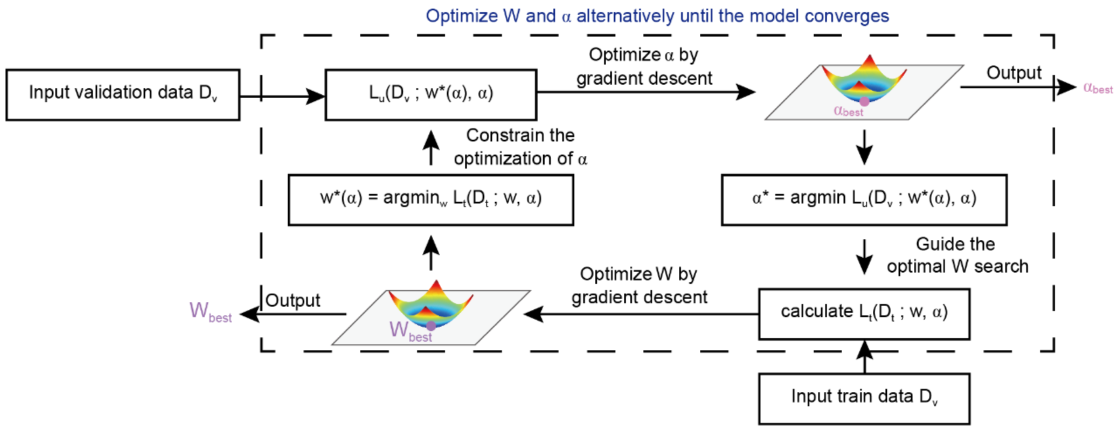

Supplementary Figure 15 | The optimization flow of HIFI.

Supplementary Tables

Supplementary Table 1 | Forward process and operational complexity breakdown of different spiking neurons.

Spiking Neuron	Step	Forward Process	Operational Complexity	Total Operational Complexity
IF	1	$I_t^n = W^{m,n}X^m$	$mn\mathrm{Fr}_{\mathrm{in}}E_{\mathrm{AC}}$	$(mn\mathrm{Fr}_{\mathrm{in}} + 2n)E_{\mathrm{AC}}$
	2	$H_t^n = V_{t-1}^n + I_t^n$	nE_{AC}	
	3	$S_t^n = \Theta(H_t^n - V_{th})$	nE_{AC}	
	4	$V_t^n = H_t^n(1 - S_t^n)$	/	
LIF	1	$I_t^n = W^{m,n}X^m$	$mn\mathrm{Fr}_{\mathrm{in}}E_{\mathrm{AC}}$	$(mn\mathrm{Fr}_{\mathrm{in}} + n)E_{\mathrm{AC}} + nE_{\mathrm{MAC}}$
	2	$H_t^n = \alpha V_{t-1}^n + I_t^n$	nE_{MAC}	
	3	$S_t^n = \Theta(H_t^n - V_{th})$	nE_{AC}	
	4	$V_t^n = H_t^n(1 - S_t^n)$	/	
PLIF	1	$I_t^n = W^{m,n}X^m$	$mn\mathrm{Fr}_{\mathrm{in}}E_{\mathrm{AC}}$	$(mn\mathrm{Fr}_{\mathrm{in}} + n)E_{\mathrm{AC}} + nE_{\mathrm{MAC}}$
	2	$H_t^n = f(\alpha)V_{t-1}^n + I_t^n$	nE_{MAC}	
	3	$S_t^n = \Theta(H_t^n - V_{th})$	nE_{AC}	
	4	$V_t^n = H_t^n(1 - S_t^n)$	/	
GLIF	1	$C_t^n = W^{m,n}X^m$	$mn\mathrm{Fr}_{\mathrm{in}}E_{\mathrm{AC}}$	$(mn\mathrm{Fr}_{\mathrm{in}} + 4n)E_{\mathrm{AC}} + 4nE_{\mathrm{MAC}}$
	2	$H_t^n = [1 - \alpha(1 - \tau_{exp})]V_{t-1}^n$	nE_{MAC}	
	3	$L_t^n = H_t^n - (1 - \alpha)\tau_{lin}$	nE_{AC}	
	4	$I_t^n = [1 - \beta(1 - g_t)]C_t^n$	nE_{MAC}	
	5	$F_t^n = -(0 + \gamma)H_t^n - (1 - \gamma)V_{re}$	$2nE_{\mathrm{MAC}}$	
	6	$V_t^n = L_t^n + I_t^n + F_t^n S_{t-1}^n$	$2nE_{\mathrm{AC}}$	
	7	$S_t^n = \Theta(H_t^n - V_{th})$	nE_{AC}	
Ours	1	$I_t^n = W^{m,n}X^m - \gamma S_{t-1}^n$	$(mn\mathrm{Fr}_{\mathrm{in}} + n)E_{\mathrm{AC}}$	$(mn\mathrm{Fr}_{\mathrm{in}} + 3n)E_{\mathrm{AC}} + 3nE_{\mathrm{MAC}}$
	2	$H_t^n = f((1 - \tau)V_{t-1}^n + \tau V_{th} + CI_t^n)$	$3nE_{\mathrm{MAC}}$	
	3	$S_t^n = \Theta(H_t^n - V_{th})$	nE_{AC}	
	4	$V_t^n = H_t^n(1 - S_t^n) + V_{re}S_t^n$	nE_{AC}	

m: input dim n: output dim $\mathrm{Fr}_{\mathrm{in}}$: input firing rate $E_{\mathrm{AC}}/E_{\mathrm{MAC}}$: the energy cost per AC/MAC operation

Supplementary Table 2 | Energy efficiency comparison of HIFI with state-of-the-art SNNs on neuromorphic devices.

Energy Efficiency Comparison on CIFAR-10

Method	Time Step	Spike (10^6)	Acc (%)	Normalized Energy	
				TrueNorth	SpiNNaker
Diet-SNN ['TNNLS2023]	5	0.6000	92.70	1.0000	1.0000
SLTT ['ICCV202]	6	0.4936	94.59	1.1720	1.1337
GLIF ['NIPS2022]	2	0.2557	94.44	0.4019	0.4046
	4	0.4546	94.85	0.7969	0.7925
	6	0.6467	95.03	1.1909	1.1785
**Ours**	**2**	**0.1871**	**95.67**	**0.3935**	**0.3845**
	**4**	**0.3485**	**95.94**	**0.7838**	**0.7614**
	**6**	**0.5555**	**95.98**	**1.1797**	**1.1518**
Underlined digits denote the best performance among baseline SNNs.					

Energy Efficiency Comparison on CIFAR-100

Method	Time Step	Spike (10^6)	Acc (%)	Normalized Energy	
				TrueNorth	SpiNNaker
Diet-SNN ['TNNLS2023]	5	0.6154	69.67	1.0000	1.0000
SLTT ['ICCV202]	6	0.3096	74.67	1.1472	1.0749
GLIF ['NIPS2022]	2	0.2790	75.48	0.4041	0.4096
	4	0.4998	77.05	0.8009	0.8022
	6	0.7254	77.35	1.1984	1.1962
**Ours**	**2**	**0.2143**	**77.80**	**0.3961**	**0.3907**
	**4**	**0.4251**	**78.76**	**0.7917**	**0.7804**
	**6**	**0.6381**	**79.32**	**1.1876**	**1.1707**
Underlined digits denote the best performance among baseline SNNs.					

Energy Efficiency Comparison on ImageNet

Method	Time Step	Spike (10^6)	Acc (%)	Normalized Energy	
				TrueNorth	SpiNNaker
Diet-SNN ['TNNLS2023]	5	30.9096	69.00	1.0000	1.0000
**Ours**	**4**	**7.2095**	**69.11**	**0.3439**	**0.2805**
Underlined digits denote the best performance among baseline SNNs.					

Supplementary Table 3 | The classification accuracy of HIFI with different degrees of freedom and SOTA SNNs on CIFAR 10 and CIFAR 100.

Method	Degrees of freedom	Time step	Accuracy
	CIFAR 10		
PLIF [ICCV2021]	1 (τ)	6	94.25%
Dspike [NIPS2021]	0	6	93.50%
DSR [CVPR2022]	1 (u_{th})	20	95.40%
GLIF [NIPS2022]	8 ($\tau_{lin}, \tau_{exp}, V_{re}, g_t, V_{th}, \alpha, \beta, \gamma$)	6	94.88%
TET [ICLR2022]	0	6	94.50%
QCFS [ICLR2022]	0	8	94.82%
SLTT [ICCV2023]	0	6	94.59%
Diet-SNN [TNNLS2023]	2 (τ, u_{th})	10	92.54%
OSR [ICLR2024]	0	4	95.20%
TAB [ICLR2024]	0	6	94.81%
	**5 ($\tau, \gamma, C, u_{th}, u_{thre}$)**	**4**	**95.94%**
**HIFI (Ours)**	**2 (τ, u_{th})**	**4**	**95.92%**
	**1 (u_{th})**	**4**	**95.85%**
	CIFAR 100		
Dspike [NIPS2021]	0	6	74.24%
DSR [CVPR2022]	1 (u_{th})	20	78.50%
GLIF [NIPS2022]	8 ($\tau_{lin}, \tau_{exp}, V_{re}, g_t, V_{th}, \alpha, \beta, \gamma$)	4	77.28%
TET [ICLR2022]	0	6	74.72%
QCFS [ICLR2022]	0	32	78.48%
SLTT [ICCV2023]	0	6	74.67%
Diet-SNN [TNNLS2023]	2 (τ, u_{th})	5	64.07%
OSR [ICLR2024]	0	4	77.86%
TAB [ICLR2024]	0	4	76.82%
	**5 ($\tau, \gamma, C, u_{th}, u_{thre}$)**	**4**	**78.76%**
**HIFI (Ours)**	**2 (τ, u_{th})**	**4**	**78.00%**
	**1 (u_{th})**	**4**	**77.80%**

Supplementary Table 4 | Comparison of HIFI with state-of-the-art SNNs on Neuromorphic Datasets.

Dataset	Method	Comments	Architecture	Accuracy
	STBP	Surrogate gradient method	3-layer-CNN	99.45%
	SNU	Surrogate gradient method	3-layer-CNN	99.30%
	TSSL-BP	Temporal backpropagation	3-layer-CNN	99.28%
N-MNIST	Sparse SCNN[22]	Surrogate gradient method	4-layer-CNN	99.38%
	LIAF-Net[23]	Surrogate gradient method	ResNet-14	99.13%
	DSNN[24]	Tandem learning	DigitNet	99.31%
	**HIFI**	**This work**	3-layer-CNN	**99.45%**
	STBP	Surrogate gradient method	5-layer-CNN	93.40%
	SNU	Surrogate gradient method	5-layer-CNN	90.97%
DVS-Gesture	TSSL-BP	Temporal backpropagation	5-layer-CNN	86.50%
	RT-TSSL-KP[25]	Temporal backpropagation	3-layer-FC	87.85%
	Sparse SCNN[22]	Surrogate gradient method	5-layer-CNN	90.15%
	**HIFI**	**This work**	5-layer-CNN	**93.75%**
	STBP	Surrogate gradient method	10-layer-CNN	89.53%
SHD	SNU	Surrogate gradient method	10-layer-CNN	91.17%
	TSSL-BP	Temporal backpropagation	10-layer-CNN	89.00%
	**HIFI**	**This work**	10-layer-CNN	**92.84%**
	STBP	Surrogate gradient method	10-layer-CNN	73.10%
SSC	SNU	Surrogate gradient method	10-layer-CNN	70.12%
	TSSL-BP	Temporal backpropagation	10-layer-CNN	68.42%
	**HIFI**	**This work**	10-layer-CNN	**76.20%**
	STBP	Surrogate gradient method	5-layer-CNN	60.50%
	STBP-tdBN[26]	Surrogate gradient method	ResNet-19	67.8%
	SNU	Surrogate gradient method	CifarNet	60.40%
DVS-Cifar10	TSSL-BP	Temporal backpropagation	CifarNet	59.90%
	LIAF-Net[23]	Surrogate gradient method	ResNet-14	70.36%
	DSNN[24]	Tandem learning	CifarNet	65.59%
	**HIFI**	**This work**	CifarNet	**71.50%**

Supplementary Table 5 | Training settings for self-inhibiting neurons on single neuron biological spike signal dataset.

Training-related parameters

**Variable**	**Value**	**Variable**	**Value**
Epochs	100	Initial Learning Rate ξ_1	0.002
Weight Decay	3×10^{-4}	Optimizer	RMSprop

Supplementary Table 6 | Training settings for self-inhibiting neurons on multiple neuron biological spike signal dataset.

Training-related parameters

Variable	Value	Variable	Value
Epochs	300	Initial Learning Rate ξ_1	0.001
Weight Decay	0	Optimizer	Adam
Time scale	2.5ms		

Supplementary Table 7 | Training settings used in HIFI on image datasets.

Network

Dataset	Network	Architecture
MNIST	Full connected	784-250-10
CIFAR10	AlexNet	96C3-256C3-P2-384C3-P2-384C3-256C3-1024-1024-10
	CIFARNet	128C3-256C3-P2-512C3-P2-1024C3-512C3-1024-512-10
	VGG9	64C3-64C3-P2-128C3-128C3-P2-256C3-256C3-256C3-P2-1024-10
	ResNet18	64C3-BasicBlock64(Stride1)*2-BasicBlock128(Stride2)*2-BasicBlock256(Stride2)*2-BasicBlock512(Stride2)*2-10
CIFAR100	ResNet18	64C3-BasicBlock64(Stride1)*2-BasicBlock128(Stride2)*2-BasicBlock256(Stride2)*2-BasicBlock512(Stride2)*2-100
ImageNet	SEW-ResNet-34	64C7(Stride2)-BasicBlock64(Stride1)*3-BasicBlock128(Stride2)*4-BasicBlock256(Stride2)*6-BasicBlock512(Stride2)*3-1000

Training-related parameters

Variable	Value	Variable	Value
Batch Size	32 / 128 / 128 / 32*	Initial Inner Learning Rate ξ_1	10^{-3} / 10^{-1} / 10^{-1} / 10^{-1}*
Epochs	50 / 1024 / 1024 / 300*	Initial Outer Learning Rate ξ_2	10^{-3}
Timesteps	5 / 2, 4, 6 / 2, 4, 6 / 4*	Inner optimizer	SGD
Weight Decay	3×10^{-4} / 5×10^{-5} / 5×10^{-4} / 0*	Outer optimizer	Adam / SGD / SGD / SGD*

* MNIST / CIFAR10 / CIFAR100 / ImageNet

Supplementary Table 8 | Training settings used in HIFI on neuromorphic datasets.

Training-related settings

**Variable**	**Value**	**Variable**	**Value**
Batch Size	64	Initial Inner Learning Rate ξ_1	10^{-3}
Epochs	100 / 200 / 100 / 100*	Initial Outer Learning Rate ξ_1	10^{-3}
Frames	10 / 20 / 5 / 5 *	Inner optimizer	Adam
Weight Decay	0 / 3×10^{-4} / 5×10^{-4} / 3×10^{-4}*	Outer optimizer	Adam

* N-MNIST / DVS128 Gesture / SHD & SSC / DVS-CIFAR10

Supplementary Table 9 | Training settings used in HIFI on scRNA-seq.

Training-related settings

Variable	Value	Variable	Value
Batch Size	128	Initial Inner Learning Rate ξ_1	10^{-3}
Epochs	200	Initial Outer Learning Rate ξ_1	5×10^{-4}
Timesteps	40	Inner optimizer	RMSprop
Weight Decay	3×10^{-4}*	Outer optimizer	Adam

Supplementary Table 10 | Default parameters used in HIFI.

Variable	Value
Membrane Decay τ	0.2 / 0.8*
Self-inhibition Factor γ	0.05
Capacitance C	0.9
Potential Threshold u_{thre}	1.0
Resting Potential u_{rest}	0
Hyper-parameter λ	0.01

* Standard Image dataset / Neuromorphic dataset

Supplementary References

1. Sinha A, Malo P, Deb K. A review on bilevel optimization: from classical to evolutionary approaches and applications. *IEEE Transactions on Evolutionary Computation*. 2017; **22**(2): 276-95.
2. Colson B, Marcotte P, Savard G. An overview of bilevel optimization. *Annals of operations research*. 2007; **153**(1): 235-56.
3. Dempe S, Zemkoho A. Bilevel optimization. *Springer optimization and its applications*: Springer; 2020.
4. Hawrylycz MJ, Lein ES, Guillozet-Bongaarts AL *et al.* An anatomically comprehensive atlas of the adult human brain transcriptome. *Nature*. 2012; **489**(7416): 391-9.
5. Gilja V, Nuyujukian P, Chestek CA *et al.* A high-performance neural prosthesis enabled by control algorithm design. *Nat Neurosci*. 2012; **15**(12): 1752-7. doi: 10.1038/nn.3265
6. Singanamalla SKR, Lin CT. Spiking Neural Network for Augmenting Electroencephalographic Data for Brain Computer Interfaces. *Front Neurosci*. 2021; **15**: 651762. doi: 10.3389/fnins.2021.651762
7. Naud R, Gerhard F, Mensi S *et al.* Improved similarity measures for small sets of spike trains. *Neural computation*. 2011; **23**(12): 3016-69.
8. Pozzorini C, Mensi S, Hagens O *et al.* Automated high-throughput characterization of single neurons by means of simplified spiking models. *PLoS computational biology*. 2015; **11**(6): e1004275.
9. LeCun Y, Bottou L, Bengio Y *et al.* Gradient-based learning applied to document recognition. *Proceedings of the IEEE*. 1998; **86**(11): 2278-324.
10. Meng Q, Xiao M, Yan S *et al.* Towards memory-and time-efficient backpropagation for training spiking neural networks. In: International Conference on Computer Vision; Paris, France. 2-6 October 2023.
11. Fang W, Yu Z, Chen Y *et al.* Deep residual learning in spiking neural networks. *Advances in Neural Information Processing Systems*. 2021; **34**: 21056-69.
12. Kundu S, Nazemi M, Pedram M *et al.* Pre-defined sparsity for low-complexity convolutional neural networks. *IEEE Transactions on Computers*. 2020; **69**(7): 1045-58.
13. Datta G, Kundu S, Beerel PA. Training energy-efficient deep spiking neural networks with single-spike hybrid input encoding. In: International Joint Conference on Neural Networks; Shenzhen, China. 18-22 July 2021.
14. Horowitz M. 1.1 computing's energy problem (and what we can do about it). In: IEEE International Solid-State Circuits Conference Digest of Technical Papers (ISSCC); San Francisco, USA. 09-13 February 2014.
15. Rathi N, Srinivasan G, Panda P *et al.* Enabling deep spiking neural networks with hybrid conversion and spike timing dependent backpropagation. *arXiv preprint arXiv:200501807*. 2020.
16. Datta G, Kundu S, Jaiswal AR *et al.* ACE-SNN: Algorithm-hardware co-design of energy-efficient & low-latency deep spiking neural networks for 3d image recognition. *Frontiers in neuroscience*. 2022; **16**: 815258.
17. Orchard G, Jayawant A, Cohen GK *et al.* Converting static image datasets to spiking neuromorphic datasets using saccades. *Frontiers in neuroscience*. 2015; **9**: 437.
18. Amir A, Taba B, Berg D *et al.* A low power, fully event-based gesture recognition system. In: IEEE Conference on Computer Vision and Pattern Recognition; Venice, Italy. 22-29 October 2017.
19. Cheng W, Luo H, Yang W *et al.* Structure-aware network for lane marker extraction with dynamic vision sensor. *arXiv preprint arXiv:200806204*. 2020.

20. Cramer B, Stradmann Y, Schemmel J *et al.* The heidelberg spiking data sets for the systematic evaluation of spiking neural networks. *IEEE Transactions on Neural Networks and Learning Systems*. 2020.

21. Wolf FA, Angerer P, Theis FJ. SCANPY: large-scale single-cell gene expression data analysis. *Genome biology*. 2018; **19**(1): 1-5.

22. Yin H, Lee JB, Kong X *et al.* Energy-Efficient Models for High-Dimensional Spike Train Classification using Sparse Spiking Neural Networks. In: The 27th ACM SIGKDD Conference on Knowledge Discovery and Data Mining; New York, USA. 14-18 August 2021.

23. Wu Z, Zhang H, Lin Y *et al.* Liaf-net: Leaky integrate and analog fire network for lightweight and efficient spatiotemporal information processing. *IEEE Transactions on Neural Networks and Learning Systems*. 2021.

24. Wu J, Chua Y, Zhang M *et al.* A tandem learning rule for effective training and rapid inference of deep spiking neural networks. *IEEE Transactions on Neural Networks and Learning Systems*. 2021.

25. Boone R, Zhang W, Li P. Efficient Biologically-Plausible Training of Spiking Neural Networks with Precise Timing. In: *International Conference on Neuromorphic Systems 2021, 2021*, p. 1-8.

26. Zheng H, Wu Y, Deng L *et al.* Going deeper with directly-trained larger spiking neural networks. In: AAAI Conference on Artificial Intelligence; Vancouver, Canada. 2-9 February 2021.
